# Graft union formation involves interactions among bud signals, carbon availability, dormancy release, wound responses and non‐self‐communication in grapevine

**DOI:** 10.1111/tpj.70244

**Published:** 2025-06-11

**Authors:** Grégoire Loupit, Josep Valls Fonayet, Joseph Tran, Virginie Garcia, Irène Hummel, Pierre Petriacq, Philippe Gallusci, Margot Berger, Céline Franc, Gilles de Revel, Nathalie Ollat, Sarah Jane Cookson

**Affiliations:** ^1^ EGFV Univ. Bordeaux, Bordeaux Sciences Agro, INRAE, ISVV F‐33882 Villenave d'Ornon France; ^2^ Bordeaux Metabolome Facility, MetaboHUB, PHENOME‐EMPHASIS 33140 Villenave d'Ornon Talence France; ^3^ Unité de recherche Œnologie, EA 4577, USC 1366 INRAE, ISVV Université de Bordeaux F33882 Villenave d'Ornon Talence France; ^4^ Université de Lorraine, AgroParisTech, INRAE, UMR Silva F‐54000 Nancy France; ^5^ Univ. Bordeaux, INRAE, UMR1332 BFP 33882 Villenave d'Ornon France; ^6^ Present address: Umeå Plant Science Centre, Department of Forest Genetics and Plant Physiology Swedish University of Agricultural Sciences Umeå Sweden

**Keywords:** wounding, WGCNA, RNAseq, *Vitis vinifera*, Scion, rootstock

## Abstract

Grafting plants uses intrinsic wound‐healing mechanisms to join together different organisms, yet the processes underpinning graft union formation remain poorly understood. To further our understanding of the molecular reprogramming triggered by grafting and wounding in a perennial plant, we characterised the transcriptome and metabolome of intact and wounded un‐grafted scions and rootstocks, and homo‐ and hetero‐grafts at 0 and 14 days after grafting/wounding in grapevine. We show that grafting triggered the coordinated activation of gene expression and the accumulation of lipids and phenolic compounds in comparison with intact tissues. We highlight an asymmetry in gene expression above and below the graft interface, which is in part not only due to carbon status, but also to intrinsic differences in gene expression between un‐grafted scions and rootstocks, and their differential responses to wounding. We found that β‐1,4‐glucanases were differentially expressed in response to both wounding and grafting and demonstrated that exogenous β‐1,4‐glucanase application increased grafting success rate. Grafting, wounding, homo‐graft and hetero‐graft‐specific transcriptome responses were characterised. The comprehensive experimental design of the dataset containing all necessary control samples allowed the identification of genes and metabolites potentially involved in wounding and grafting responses in an iconic grafted fruit crop. This is important because knowledge of genes regulating graft union formation could be leveraged for the selection of new, highly graft‐compatible cultivars in the future.

## Significance Statement

Grafting is essential to the cultivation of many perennial fruit crops, but we have little understanding of the processes involved. Here, we identify wounding and grafting‐specific gene expression and metabolite accumulation profiles and identify some of the factors regulating these changes.

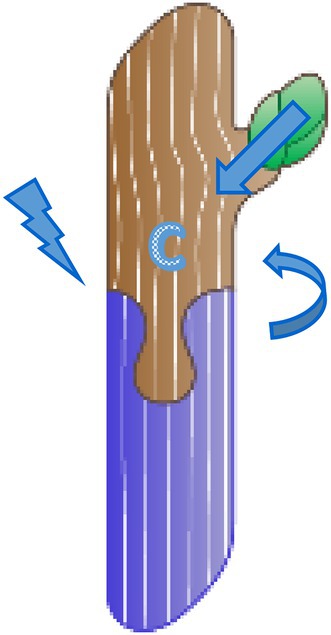

## INTRODUCTION

Grafting is widely used in agriculture to combine the characteristics of two genetically different plants in one organism (Mudge et al., 2009). The molecular mechanisms underlying graft union formation are becoming increasingly well understood, and the range of genotypes that can be grafted together has expanded (as reviewed by Loupit, Brocard, et al. (2023); Feng, Augstein, et al. (2024)). In the context of climate change and new emerging diseases/pests, there is much interest in grafting and selecting new rootstock genotypes with improved stress tolerance.

During graft union formation, there is generally the proliferation of undifferentiated cells to form a callus and then the vascular tissues connect between the two genotypes. The changes in gene expression underlying graft union formation have been characterised in a range of plant species, from the model plant Arabidopsis (Melnyk et al., 2018; Yin et al., 2012) to herbaceous (Xie et al., 2019; Xie et al., 2022) and perennial crop species. The transcriptional response to grafting generally integrates wound responses, gene activation for callus formation, regulation of cell wall‐related genes and gene expression for cambial and vascular tissue development (as reviewed by Loupit, Brocard, et al. (2023); Feng, Augstein, et al. (2024)). Many cell wall modifications occur during graft union formation; for example, pectin‐rich material accumulates at the graft interface, which presumably has a role in adhering the scion and rootstock together (Jeffree & Yeoman, 1983). In addition, modifying β‐1,4‐glucanase activity can impact tissue adhesion and grafting success. Notaguchi et al. (2020) identified the β‐1,4‐glucanase GH9B3, which enhances tissue adhesion and improves grafting success in tobacco. Moreover, Kawakatsu et al. (2020) demonstrated that exogenous cellulase application promotes tissue adhesion in in vitro stem unions, while xylem connectivity is delayed in the β‐1,4‐glucanase mutant *korrigan1/kor1* (Zhang et al., 2022).

Complex cascades of transcription factor interactions regulate different aspects of wound‐healing mechanisms, callus formation and the development of vascular tissues (as reviewed by Feng, Augstein, et al. (2024)). For example, in Arabidopsis, the ETHYLENE RESPONSE FACTOR/APETALA2 (ERF/AP2) transcription factor *WOUND‐INDUCED DEDIFFERENTIATION1/WIND1* is rapidly induced in response to wounding, and overexpression of *WIND1* induces callus cell formation (Iwase et al., 2011). WIND1 controls the expression of various transcription factors that regulate wound‐induced callus formation and shoot regeneration, such as the ERF transcription factor *ENHANCER OF SHOOT REGENERATION1/ESR1* (Iwase et al., 2017). Moreover, *WIND1* expression is potentially regulated by another ERF transcription factor, ERF115 (Heyman et al., 2016), a wound‐inducible regulator of stem cell regeneration that interacts with auxin signalling (Canher et al., 2020).

Auxin, coming from the shoot apex, is essential for tissue adhesion, cell proliferation and vascular formation, which could explain why there is a more rapid xylem and phloem formation above than below the graft interface (Melnyk et al., 2018; Serivichyaswat et al., 2024). It has been suggested that during graft union formation, auxin drives many changes in gene expression, including some of the asymmetric transcript accumulation responses above and below the graft interface (Melnyk et al., 2018). The asymmetry of gene expression is seen both across wound sites (Asahina et al., 2011) and graft interfaces of Arabidopsis (Melnyk et al., 2018), tomato (Xie et al., 2019) and pecan (Mo et al., 2024). Furthermore, in grafts made with actively growing and photosynthesising tissues such as hypocotyl grafts of Arabidopsis, sugars accumulate in scion tissues, that is, above the graft interface, leading to a local increase in the expression of genes associated with sugar responses (Melnyk et al., 2018). It is also possible that the response to wounding is different between scions and rootstocks (tissues with and without a bud, respectively), which could be an additional factor underlying the asymmetry of gene expression above and below the graft interface.

The accumulation of metabolites at the graft interface has been studied in many species (as reviewed by Loupit and Cookson (2020)). For example, in grapevine, 1 month after grafting, primary metabolites such as glucose, glutamine and γ‐aminobutyric acid accumulate at the graft interface, along with increased phenylalanine lyase activity, which is associated with decreased phenylalanine concentration (Prodhomme et al., 2019). Concordantly, many polyphenols accumulate at the graft interface of grapevine (Loupit, Fonayet, et al., 2023), in particular stilbenes in the damaged xylem parenchyma and naringenin in the newly formed callus tissues (Loupit, Fonayet, et al., 2023). In addition, the induction of *AtWIND1* in tobacco modifies the concentration of many metabolites, such as increasing the concentration of fructose, proline, γ‐aminobutyric acid and trehalose‐6‐phosphate (Iwase et al., 2018), highlighting the importance of metabolite changes for callus formation.

Generally, in perennial plants, over‐wintering stems are grafted just before the end of the winter. The material used is therefore ecodormant, which implies that mechanisms of graft union formation and spring‐induced dormancy release occur in parallel (Cookson et al., 2013). As such, graft union formation in perennial plants may be quite different from that of annuals. For example, since there are no actively photosynthesising tissues used for grapevine grafting and woody canes of grapevine are rich in carbohydrates, it seems likely that few asymmetric gene expression patterns across the graft interface would be associated with differences in sugar accumulation.

The goal of this study was to characterise transcriptome and metabolome responses to grafting of a woody, perennial plant, as well as the molecular events triggered by grafting two different genotypes together. To strengthen our understanding of the molecular events occurring during the graft union formation, we developed a comprehensive experimental design, encompassing numerous and necessary control samples, including intact scions and rootstocks, wounded scions and rootstocks, as well as grafted scions and rootstocks of different scion/rootstock combinations. The dataset allows us to ask previously unanswered questions about grafting and wounding responses in plants. For instance, do un‐grafted rootstocks (without buds) and scions (with buds) respond differently to wounding? Which genes are asymmetrically expressed in response to wounding and grafting in perennial, woody tissues? How does this asymmetry relate to the presence of a bud? How do responses to wounding and grafting compare when they are done on the same tissues? Does grafting with a non‐self‐partner modify gene expression in the early stages of graft union formation?

## RESULTS

**Figure 1:**
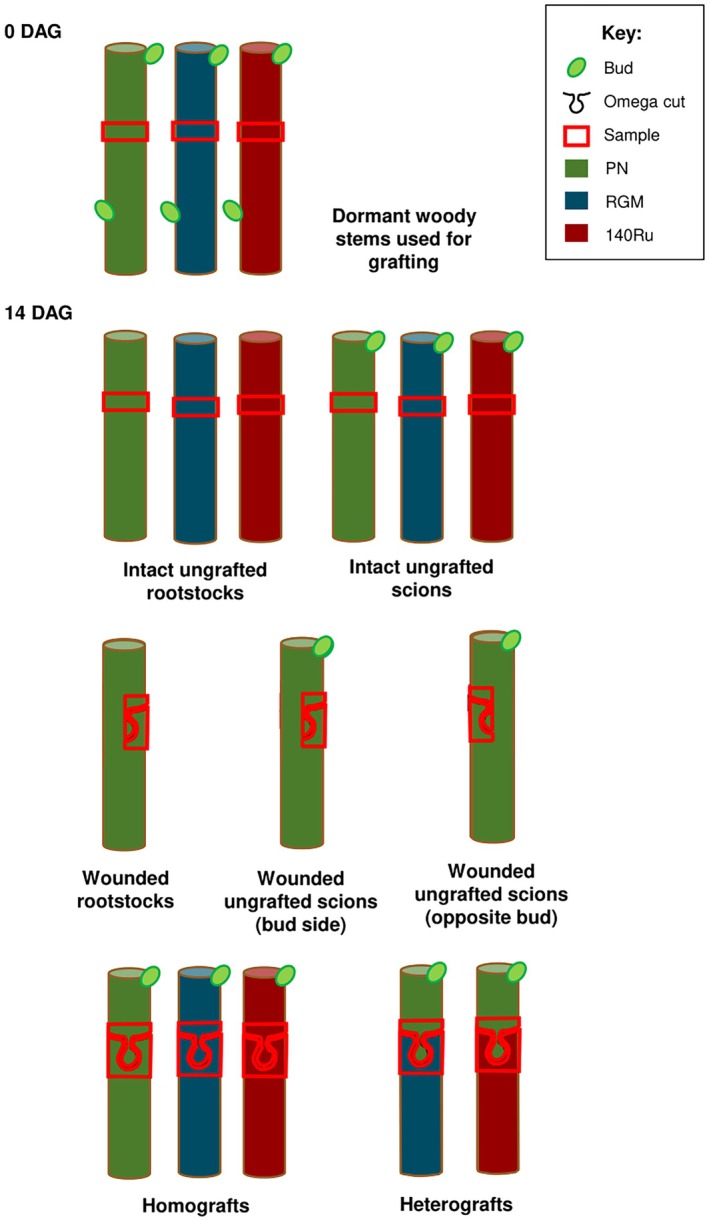
Experimental design. Samples were taken from dormant, overwintering stems (0 days after grafting, DAG), and 14 days later (14 DAG) from intact, un‐grafted scions and rootstocks (with and without buds, respectively), wounded rootstocks (without buds) and scions (wounded under and opposite the bud), and homo‐ and hetero‐grafts. In wounded or grafted tissues, samples were taken separately from above and below the wound site/graft interface. Key: PN, *V. vinifera* cv. Pinot Noir; RGM, *V. riparia* cv. Gloire de Montpellier; 140Ru, *V. rupestris × V. berlandieri* cv. 140 Ruggeri.

We chose to study the metabolome and transcriptome at 0 and 14 days after grafting/wounding (DAG) because callus proliferation at the graft interface begins at approximately 14 DAG in grapevine. The experimental design of this study comprised intact and wounded un‐grafted scions (cuttings with buds) and rootstocks (cuttings without buds), and homo‐ and hetero‐grafts (Figure 1; Data S1). Firstly, we wanted to identify which factors group together the different samples; then we determined relationships between the transcripts and metabolites associated with graft union formation. Secondly, we used the RNAseq and metabolome data to ask specific questions about wounding responses and graft union formation, specifically targeting (1) which genes are differentially expressed (DE) in response to the spring‐induced dormancy release and how they relate to grafting‐responsive genes, (2) how the presence of a bud modifies the transcriptomic and metabolic response to wounding (grafts generally combine scions that have buds with rootstocks that do not), (3) how the asymmetric responses above and below wound and graft sites compare when studied in the same experimental system, (4) which genes are specifically expressed and which metabolites are accumulated in response to grafting in comparison to wounding, (5) whether β‐1,4‐glucanases are involved in graft union formation in grapevine and (6) whether grafting two different genotypes together induces transcriptomic and metabolic changes.

### The weighted gene co‐expression network analysis identifies modules associated with the different treatments studied

**Figure 2:**
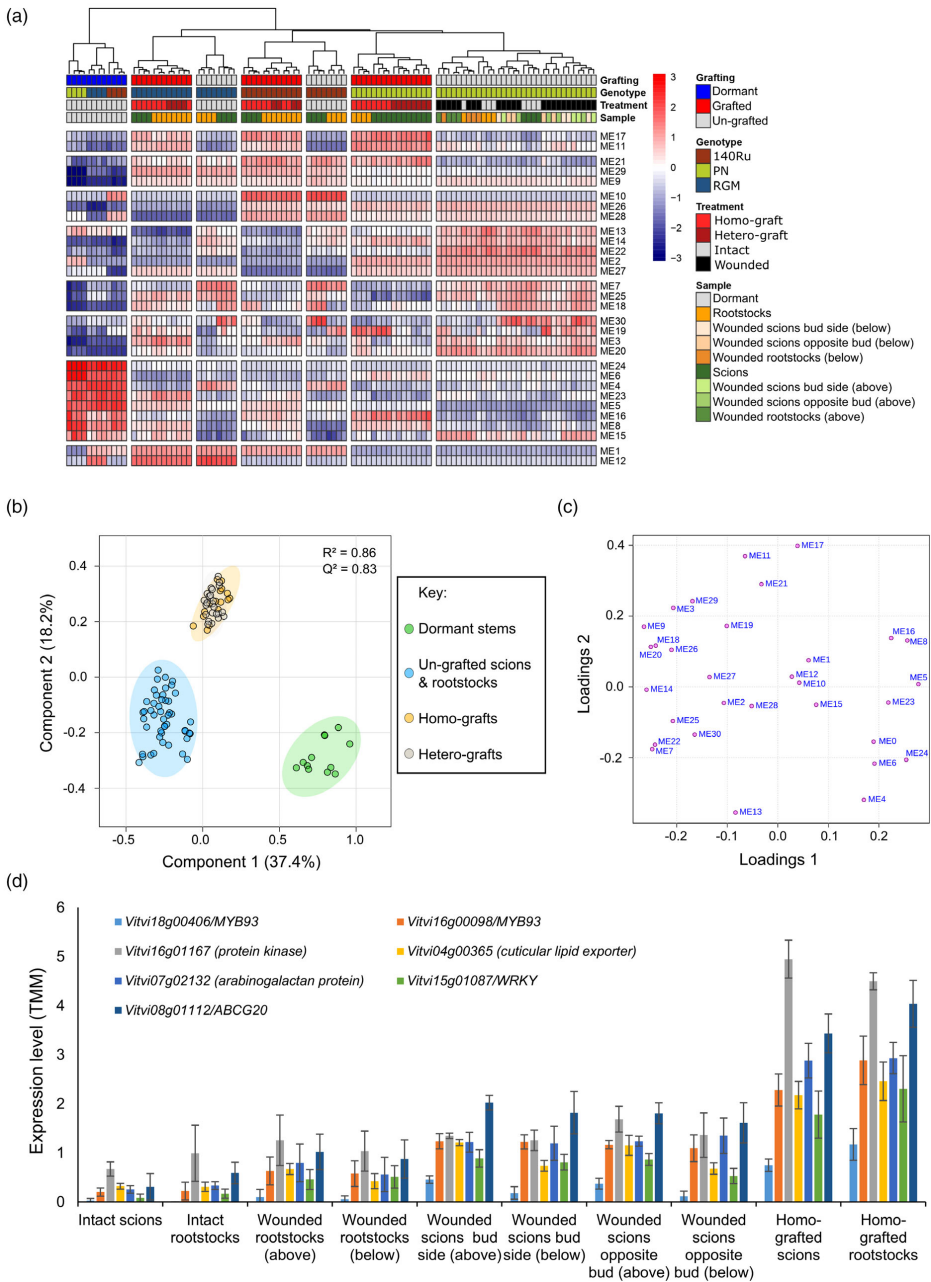
Identification of grafting and wounding‐ and grafting‐specific co‐expressed genes. (a) Heatmap of module eigengene values for all samples with hierarchical clustering; (b) Scores and (c) loading plots of Partial Least‐Squares Discriminant Analysis on module eigengene (ME) values for all samples; (d) expression profiles of the seven hub genes identified in ME17 in intact, wounded and homo‐grafted *V. vinifera* cv. Pinot noir. Key: ABCG20, ABC transporter G family 20.

The matrix of expression profiles (29 983 genes) was summarised in 30 modules of co‐expressed genes (Data S2 and S3). Gene expression profiles in the 30 modules, represented by module eigengenes (ME1‐30), was highly structured according to treatments (Figure 2a). MEs associated with specific treatments were further identified using a Partial Least‐Squares Discriminant Analysis (PLS‐DA) approach (Figure 2b). Both the dendrogram of sample‐ME relationships (Figure 2a) and the PLS‐DA scores plot (Figure 2b) separated the dormant woody stem samples taken just before grafting/wounding preparation (0 DAG) from samples harvested 14 days later (14 DAG) as demonstrated by good validation parameters of PLS‐DA (*R* ^2^ = 0.86, *Q* ^2^ = 0.83). Modules such as ME24 and ME9 were positively correlated with 0 and 14 DAG, respectively (Figure S1A,B respectively), ME11 was positively correlated with grafts and un‐grafted scions and rootstocks (Figure S1C), and grafts were positively correlated with ME17 (Figure S1D).

ME17 (447 genes) corresponded to the group of genes upregulated specifically during grafting (Figure 2a; Figure S1D). Gene ontology (GO) term and MapMan BIN enrichment analysis of the genes in ME17 showed that genes related to lipid metabolism, cutin and suberin, transport, response to abscisic acid, dephosphorylation and hydrogen peroxide catabolic processes were enriched (Tables S1 and S2, Data S3). Seven hub genes (with scaled intramodular connectivities >0.9) were identified in ME17 (Figure 1d) including two *MYB93* transcription factors and two *ABC TRANSPORTER G FAMILY/ABCG* members (*ABCG20* and *ABCG32*) (Data S3). The orthologue of the *MYB93/Vitvi18g00406* is *MYB93/AT1G34670*, a negative regulator of lateral root development that also regulates the expression of cell wall organisation genes (Uemura et al., 2023). Several genes involved in cell wall organisation were present in ME17, and two orthologues of 96 genes up‐regulated in *myb93* mutants of Arabidopsis were also present in ME17 (Uemura et al., 2023) (Data S3). The orthologue of *ABCG20/Vitvi08g01112* is *ABCG20/JAT4/AT3G53510* that is involved in regulating jasmonate transport (Li et al., 2020) and suberin formation (Yadav et al., 2014); nine genes involved in cutin and suberin formation and four genes involved in jasmonic acid action were found in ME17 (Data S3). ME17 also contains the grapevine orthologue of *ERF115/ERF114* (*Vitvi17g00025*) which is known to regulate callus formation in Arabidopsis (Heyman et al., 2016), and four LBD transcription factors, which are orthologues of genes regulating lateral root formation, growth and maintenance of cambial cells in Arabidopsis (Data S3).

ME11 contained 610 genes potentially involved in wounding and grafting (Figure 2a; Figure S1C), and this gene list was enriched in the GO terms response to oxidative stress, cell wall biogenesis, receptor‐like kinases and negative regulation of endopeptidase activity/protease inhibitors, and the MapMan BINS respiration, hormone action (particularly auxin and salicylic acid) and phenolic metabolism (Tables S3 and S4). Nine potential hub genes were identified in ME11, including a *WRKY* transcription factor, *Vitvi12g01676* (Data S3, Figure S2), which is an orthologue of *WRKY9/AT1G68150* that regulates suberin formation via cytochrome P450 genes *CYP94B3/AT3G48520* and *CYP86B1/AT5G23190* (Krishnamurthy et al., 2021). The grapevine orthologue of *CYP86B1* (*Vitvi01g00162*) was also in ME11 (Data S3, Figure S2). The hub genes of ME11 also contained *MYB102/Vitvi19g00306* (Data S3, Figure S2), previously identified in microarray experiments on genes expressed during grafting (Cookson et al., 2013). In Arabidopsis, MYB102 is involved in osmotic and wound stress responses; it is regulated by auxin and activates ethylene biosynthesis via 1‐aminocyclopropane‐1‐carboxylate (ACC) synthase, and the grapevine orthologue of *ACC* synthase (*Vitvi06g00518*) was also in ME11 (Data S3, Figure S2).

### Integration of targeted and untargeted metabolic analyses with WGCNA

To explore the relationship between transcripts and metabolites accumulated in response to the treatments studied, we investigated the correlations between the WGCNA MEs and metabolite concentrations (Figure S3). We found a strong significant positive correlation (0.73) between ME28 (which separated RGM from the other two genotypes, Figure 2a and Figure S1E) and the sum of flavanols (Figure S3). In addition, we also found a strong significant positive correlation between ME11 and ME17 with the sum of stilbenes (0.8 and 0.7, respectively) (Figure S3). The MapMan BIN related to phenolics (particularly flavonoid biosynthesis) was enriched in the genes in ME11 (Table S2); however, no genes related to stilbene synthesis were found in ME17 (Data S3).

**Figure 3:**
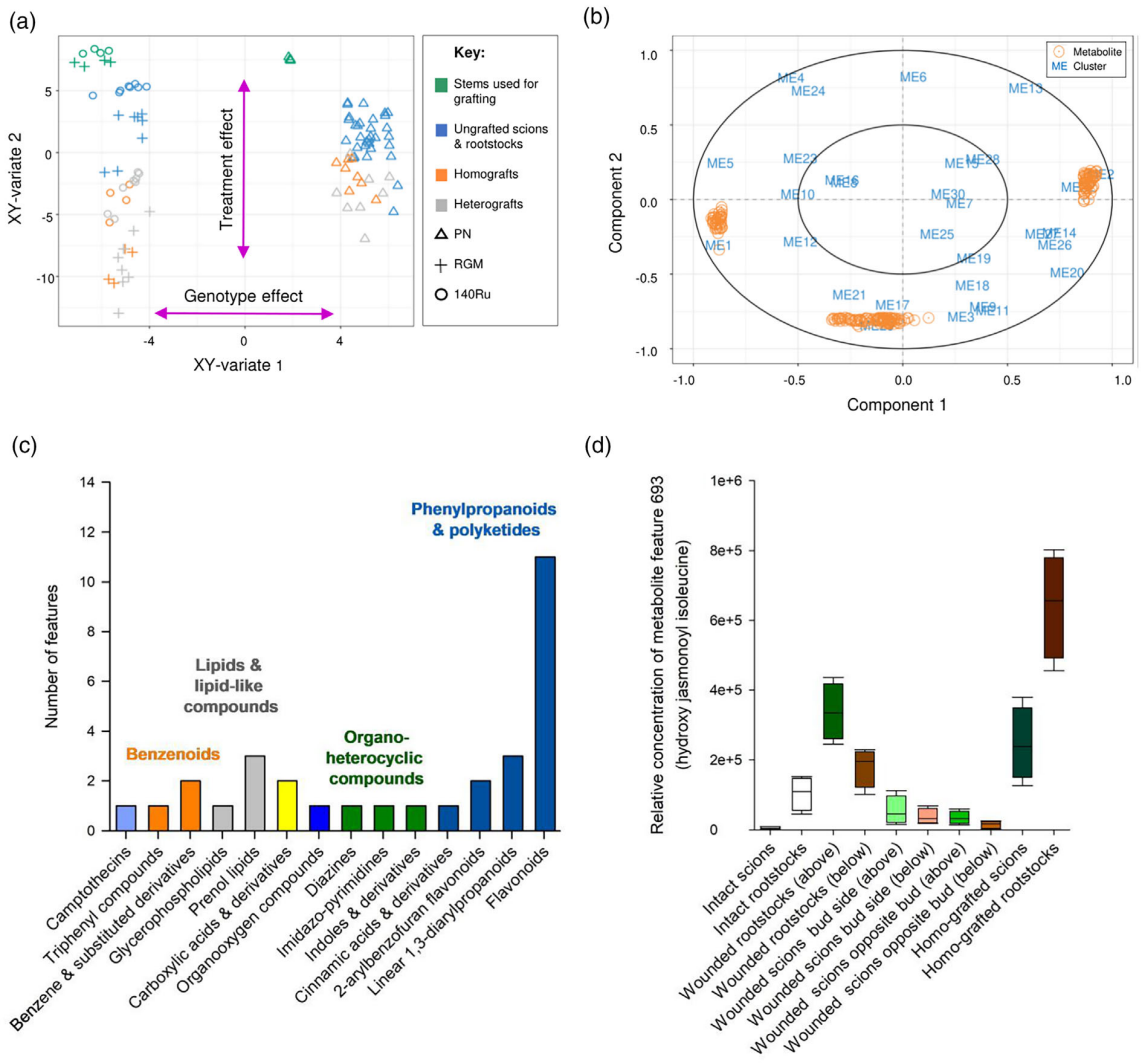
Sparse Partial Least Squares (sPLS) analysis of module eigengene and untargeted metabolic data. (a) Sample plot, arrows indicating the separation of samples according to genotype and treatment; (b) Correlation circle plot from sPLS performed on RNAseq (module eigengenes/MEs 1–30) and untargeted metabolic data; (c) Histogram of the classes of 31 features annotated of 100 extracted from component 2 in sPLS of the correlation circle, where appropriate bars coloured according to superclass; (d) the relative concentration of metabolite feature 693, corresponding to hydroxy jasmonoyl isoleucine, in intact, wounded and grafted scions and rootstocks of *V. vinifera* cv. Pinot noir. Key: PN, *V. vinifera* cv. Pinot noir; 140Ru, *V. rupestris × V. berlandieri* cv. 140 Ruggeri; RGM, *V. riparia* cv. Gloire de Montpellier.

Then, a Sparse Partial Least Squares (sPLS) approach was performed to assess correlation degrees between MEs and untargeted metabolic features. For model construction, the optimal number of principal components kept in the sPLS model was two (Figure S4), while we chose to keep ME1‐30 and 100 features for untargeted metabolome data for each component as variables in the model (Figure 3a,b). By plotting samples for the sPLS model performed on MEs and untargeted metabolome data (projection corresponds to the average components of both datasets), we observed a strong structuration according to genotype (Figure 3a). On the first axis, RGM and 140Ru samples clearly separated from PN samples (Figure 3a). Meanwhile, the sample loading on the second axis highlighted a separation gradient according to the treatment from dormant stems at grafting (0 DAG), un‐grafted scions and rootstocks, to grafts (14 DAG) (Figure 3a). We then plotted MEs and metabolites as a correlation circle plot; this revealed the relationship between certain modules and metabolite features (Figure 3b). In agreement with the sample plot (Figure 3a), the modules positively associated with grafting (such as ME17, ME11, ME29, ME9 and ME21) were located towards the negative side of component 2. As a consequence, the metabolite features plotted on the correlation circle on the negative side of component 2 corresponded to metabolites accumulated at the graft interface (Figure 3b). Then, we extracted features list from the negative side of component 2 and identified which metabolic superclass and class they belong to (Figure 3c). We were able to annotate 31 features out of 100, where 17 features belonged to phenylpropanoids and polyketides class (Figure 3c; Data S4). We identified several flavonoid and terpene glycosides, one stilbene (*cis*‐miyabenol C), lipids and lipid‐like molecules as well as a range of other molecules (Data S4). The accumulation of phenolic compounds at the graft interface is in agreement with the enrichment of MapMan BINS related to phenolics in ME11, and the accumulation of lipids and lipid‐like molecules in the graft interface agrees with the enrichment of functional categories related to lipid synthesis in ME17. This coordinated accumulation of metabolites and transcripts encoding genes of their synthesis can be seen by the visualisation of individual metabolites (such as resveratrol measured by targeted and untargeted techniques, Figure S5A/Data S5, and Figure S5B/Data S4, respectively) and transcripts (stilbene synthase *Vitvi16g04346*, Figure S5C/Data S6).

In addition to phenolic compounds, other molecules such as hydroxy jasmonoyl isoleucine accumulate at the graft interface (Figure 3d, Data S4); this compound has been identified as an active jasmonate signal that contributes to wound and defence responses in plants (Poudel et al., 2019). The accumulation of an active jasmonate signal at the graft interface is in agreement with the presence of genes related to jasmonate signalling in ME17. Taken together, omics results indicate that grafting and wounding trigger coordinated changes in both gene expression and metabolite accumulation, and that there seems to be a high degree of conservation of key transcriptome responses to wounding and grafting.

### The transition from dormancy to active growth in the spring is associated with large transcriptome changes in woody stems

To identify genes responding to spring‐induced dormancy release, we compared genes differentially expressed (DE) between dormant woody stem samples at 0 DAG and intact un‐grafted scions harvested at 14 DAG in all three genotypes and found that thousands of genes were DE (Figure S6A, Data S7). Overall, spring‐induced dormancy release was associated with the activation of gene expression and a large number of DE gene patterns common to all three genotypes (Figure S6A). Despite huge gene expression modifications between 0 and 14 DAG, we found moderate metabolome changes across the three genotypes in the untargeted metabolite analysis and little conservation in the metabolite response, with only seven metabolites accumulated in a similar fashion in all three genotypes (Figure S6B).

### There is considerable overlap between the genes DE during the transition from dormancy to active growth and those DE in response to grafting

As perennial fruit crops are often grafted in the springtime, graft union formation and spring‐induced dormancy release occur in parallel. The comparison of genes DE between grafted and intact scions and rootstocks, and those genes differentially expressed from 0 to 14 DAG shows that there is considerable overlap (Figure S7, Data S7). In PN, 420 of the 611 genes up‐regulated in response to grafting in both scions and rootstocks were also up‐regulated from 0 to 14 DAG in intact un‐grafted scions (Figure S7A); similarly, 444 of the 999 genes down‐regulated in response to grafting in scions and rootstocks were down‐regulated from 0 to 14 DAG in intact un‐grafted scions (Figure S7B).

### Un‐grafted scions and rootstocks respond differently to wounding

The presence of the bud on an overwintering woody cutting modified gene expression 14 days after the transfer to warm conditions (Data S8). For example, in PN, 474 genes were more highly expressed in intact cuttings with a bud (i.e. scions) than in cuttings without a bud (i.e. rootstocks), and 103 genes were more highly expressed in rootstocks than in scions (Data S8).

**Figure 4:**
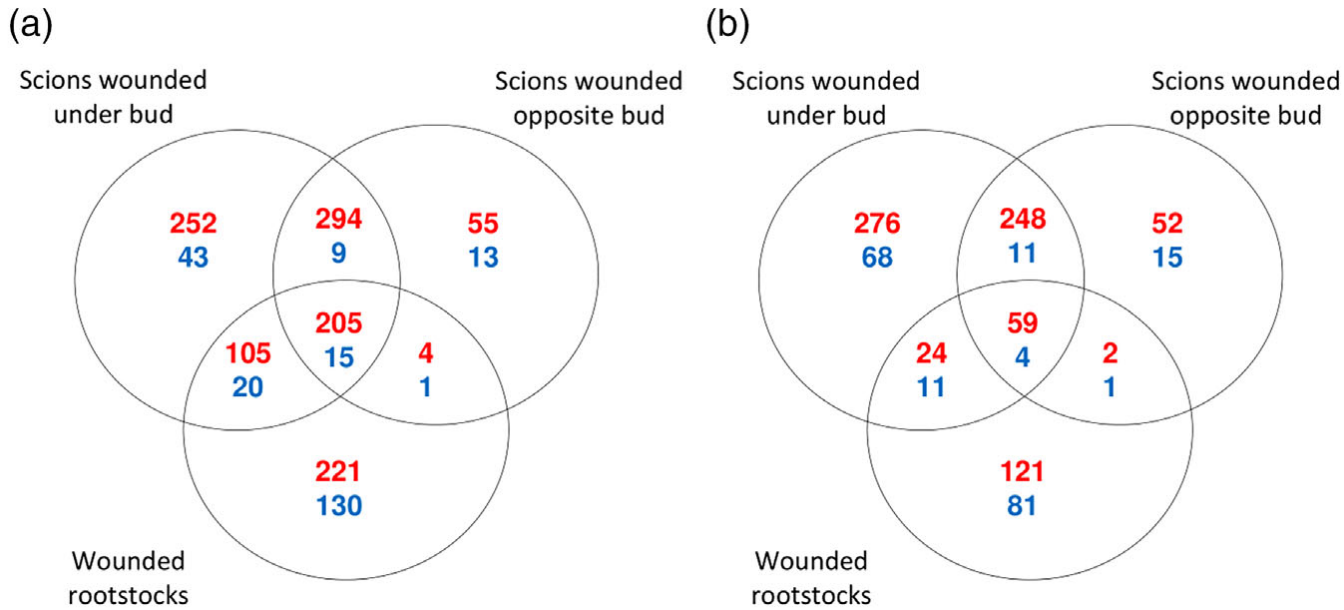
The presence of a bud modifies stem gene expression and wounding responses. Venn diagrams of the number of genes up‐ (red) and down‐ (blue) regulated in response to wounding scions on and opposite the bud side and rootstocks (without buds), (a) above and (b) below the wound site in comparison with corresponding intact tissues.

In addition to the intrinsic transcriptome differences between intact un‐grafted scions and rootstocks (i.e. un‐grafted cuttings with or without buds), we also wanted to compare their transcriptome responses to wounding. In the case of scions, we tested whether wound responses depend on the position of the wounding site in respect to bud position (Figure 4a,b, Data S9). Generally, the expression of more genes were up‐ than down‐regulated in response to wounding (Figure 4a,b). The presence of a bud had a large influence on wounding responses; for example, wounding scions on the bud side resulted in the up‐regulation of 856 genes above the wound (Figure 4a), whereas wounding rootstocks (without buds) resulted in the up‐regulation of only 535 genes above the wound (Figure 4a). The presence of a bud also modified the metabolome response to wounding (Figure S8). Interestingly, the concentration of resveratrol, a metabolite often accumulated in response to wounding in grapevine, was not affected by the presence of a bud in both the untargeted and targeted analyses (Figure S5A,B).

### There is an asymmetric response to grafting above and below the graft interface in terms of both gene expression and metabolite profile

**Figure 5:**
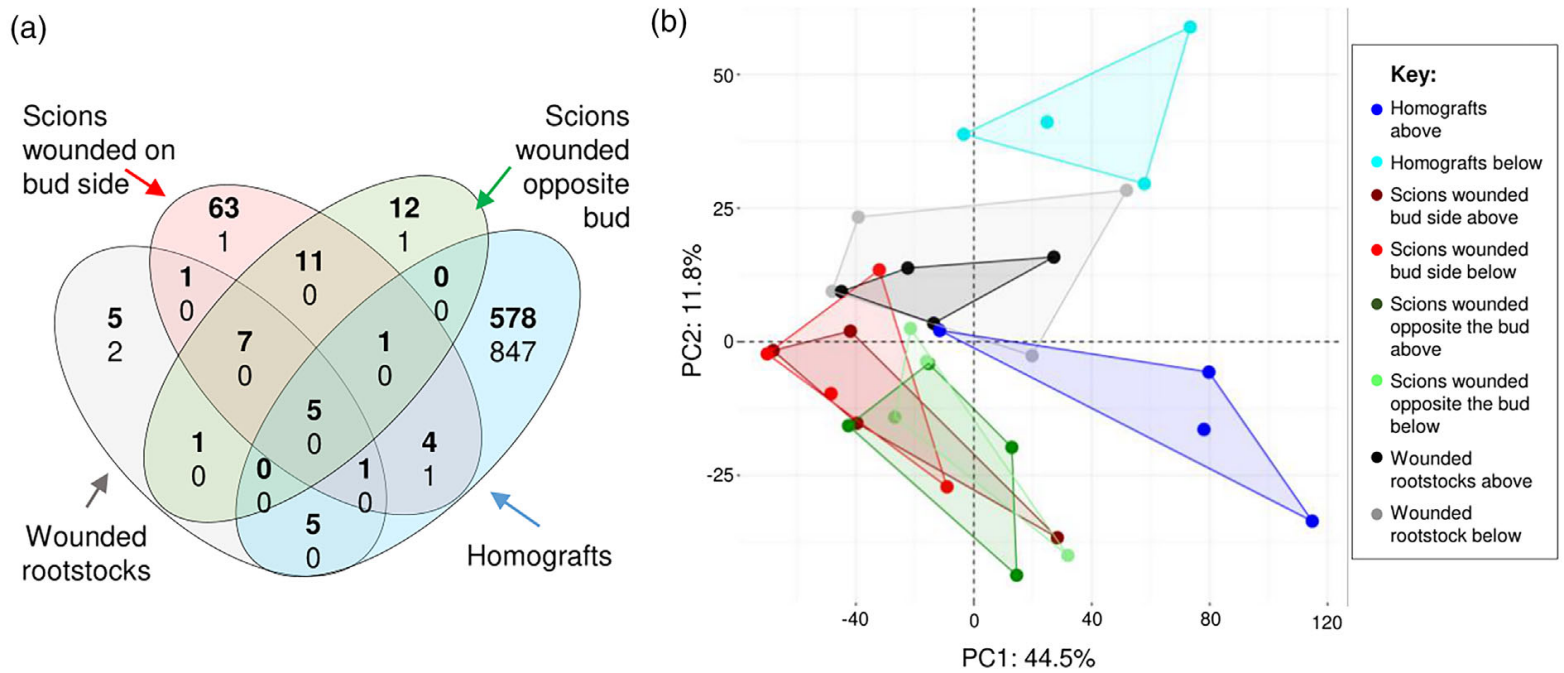
Asymmetric wounding and grafting responses above and below the wound site/graft interface. (a) Venn diagram of the number of genes more highly expressed above (bold) and below (normal text) the wound site/graft interface of rootstocks (without buds), and scions wounded either on or opposite the bud side, and homo‐grafts of *Vitis vinifera* cv. Pinot noir; (b) Principal component (PC) analysis of non‐targeted metabolites accumulated in response to wounding/grafting.

To determine whether the asymmetric responses across the graft interface or across the wound site described in other species also occur in woody grafts of grapevine, the transcriptome below and above the wound site or the graft interface was studied in un‐grafted wounded rootstocks, in un‐grafted scions wounded on the bud side and on the opposite side, as well as in PN, RGM and 140Ru homo‐grafts (Figure 5a; Figure S9A). In grapevine, scions with axillary buds are grafted; these buds potentially supply auxin heterogeneously to the graft interface, so we compared the response to wounding both under the bud and opposite the bud. As one might expect, the asymmetric transcriptome response was stronger across the graft interface than across the wound site (Figure 5a; Data S10). In agreement with the hypothesis that auxin from the shoot apex drives this asymmetric response, few genes were asymmetrically expressed across the wound site in un‐grafted rootstocks (27 genes); slightly more DE genes were found when scions were wounded on the opposite side from the bud (37 genes), whereas wounds underneath the bud triggered the DE of 95 genes across the wound site (Figure 5a). In comparison, hundreds of genes were asymmetrically expressed across the graft interface of the homo‐grafts (i.e. between the scion and rootstocks, Figure 5a; Figure S9A, Data S10).

By focusing PN homo‐grafts, we found that more genes were more highly expressed in the rootstock (848 genes) than the scion (594 genes) (Figures 5a and S9A). The genes more highly expressed in the rootstock were enriched in the MapMan BINS related to cell division, cell wall, chromatin and cytoskeleton organisation (Table S5). Whereas the 594 genes more highly expressed in the scion than the rootstock were enriched in the MapMan BINS related to carbohydrate metabolism, cell wall organisation, hormone action (particularly auxin and cytokinin) and solute transport (Table S6). This asymmetry has been at least in part ascribed to differences in carbon and auxin concentrations above and below the graft interface in Arabidopsis hypocotyl grafts (Melnyk et al., 2018). Therefore, we compared the genes expressed between the scion and rootstock with genes responding to carbon starvation in grapevine suspension cells (Berger et al., 2024) (Figure S10A). Interestingly, there was considerable overlap (305 genes) between the genes more highly expressed in the rootstock than the scion of PN/PN homo‐grafts (848 genes) and the genes repressed by carbon starvation (1663 genes) (Figure S10A). We also found considerable overlap (98 genes) between the genes more highly expressed in the scion than the rootstock of PN/PN homo‐grafts (594 genes) and those genes more highly expressed between intact scions than rootstocks (474 genes), that is, a high level of overlap between genes responding positively to the presence of a bud and genes more highly expressed above than below the graft interface (Figure S10B). We also determined to what extent the asymmetry of gene expression between the scion and rootstock in PN/PN homo‐grafts is due to differences in the wound responses of un‐grafted scions (with buds) and rootstock (without buds). We found considerable overlap between the genes more highly expressed in the rootstock than the scion of PN/PN homo‐grafts and the genes up‐regulated in response to wounding in the PN rootstocks (Figure S10C,D).

In wounded un‐grafted scions and rootstocks, the gene expression differences between above and below the wound site were not associated with differences in metabolite composition (Figure S11). However, in homo‐grafts, there was a tendency that features with annotations that accumulated preferentially above the graft interface were dipeptides or amino acids and sugar derivatives, while features with annotations that accumulated preferentially below the graft interface were secondary metabolites, for example, α‐viniferin, angoroside A, epigallocatechin‐3‐monogallate or malonyldaidzin (Figure S9B).

### Wound‐ and graft‐specific gene expression profiles

**Figure 6:**
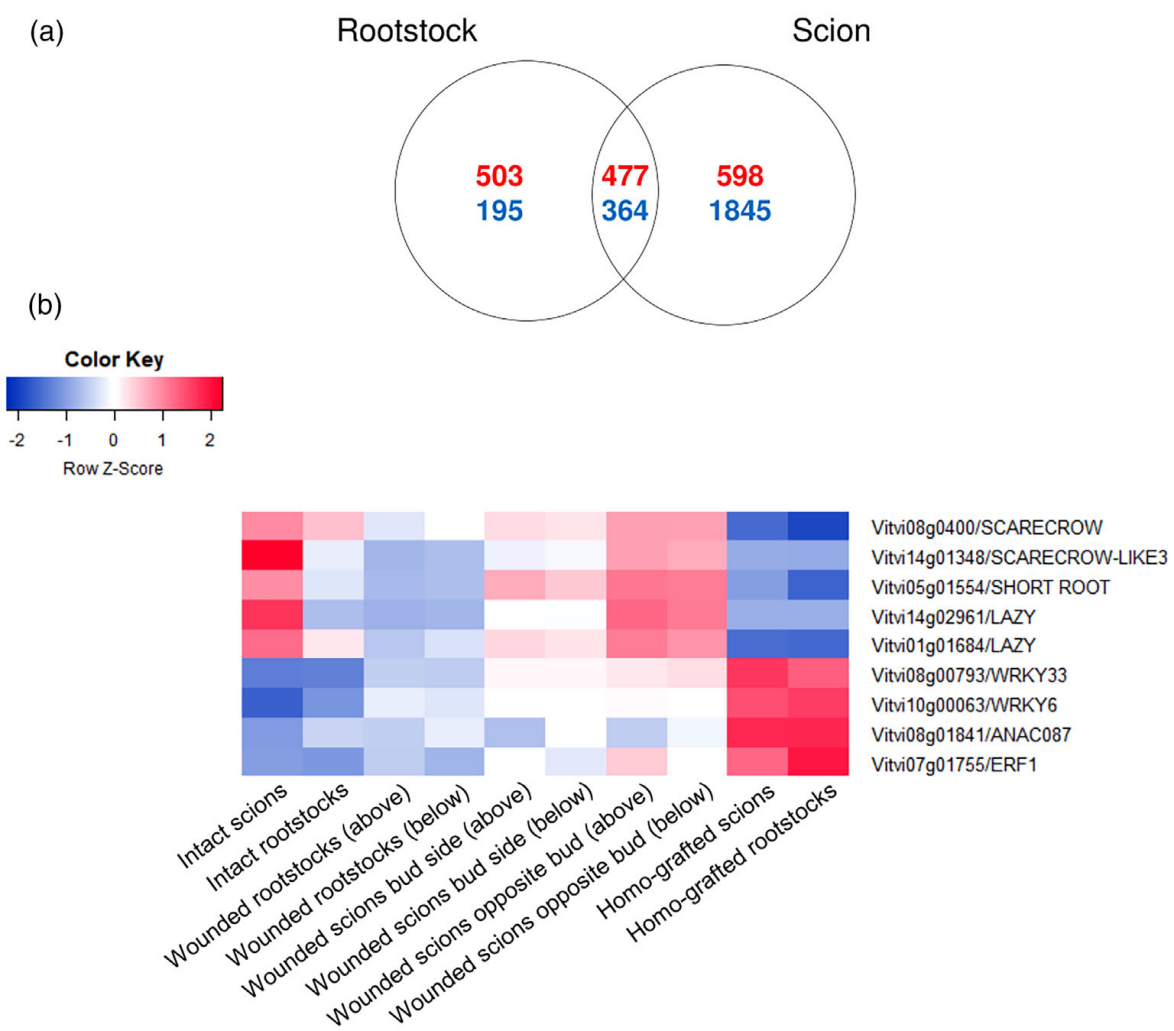
Graft and wound‐specific gene expression responses. (a) Venn diagram of the number of genes up‐ (red) and down‐regulated (blue) in response to grafting compared to wounding in rootstocks and scions; (b) heatmap of the expression of key grafting and wounding responsive genes in intact, wounded and homo‐grafted *V. vinifera* cv. Pinot noir.

To identify wound and graft‐specific gene expression profiles, we compared the genes DE between homo‐grafted and wounded PN rootstocks and scions (in the case of the scions both samples wounded under and opposite the bud were considered together in a general linear model, Data S11, Figure 6). More genes were DE between wounded and homo‐grafted scions than rootstocks, in particular for the genes more highly expressed in response to wounding than grafting (Figure 6). The 364 genes more highly expressed in response to wounding than grafting in both the scion and rootstock were enriched in the MapMan BINS related to transport, cell wall organisation, and gravity sensing and signalling (Table S7). The genes involved in trophism include orthologues of the Arabidopsis genes *SCARECROW/SCR/AT3G54220* (*Vitvi08g04002*), *SCARECROW‐LIKE PROTEIN 3* (*Vitvi14g01348*), *SHORT ROOT/SHR* (*Vitvi05g01554*) and two LAZY gravity signalling proteins (*Vitvi14g02961* and *Vitvi01g01684*). Both SCR and SHR are involved in callus formation (Zhai et al., 2023), cell division orientation (Winter et al., 2024) and protoxylem to metaxylem identity (Růžička et al., 2015) (Data S11).

The MapMan BINS enriched in the 477 genes more highly expressed in grafts than in wounded tissue in both the scion and rootstock were cell wall organisation (particularly cutin and suberin), jasmonic acid, WRKY transcription factors, protease inhibitor, PPM/PP2C Mn/Mg‐dependent phosphatases and solute transport (Table S8). The WRKY transcription factors more highly expressed in response to grafting than wounding included the grapevine orthologues of *WRKY9/AT1G68150* (*Vitvi12g01676*) which controls suberin deposition (Krishnamurthy et al., 2021), and *WRKY33*/*AT2G38470* (*Vitvi08g00793*) and *WRKY6*/*AT1G62300* (*Vitvi10g00063*) (Data S11). This gene list also included the grapevine orthologues of the ERF transcription factors *ANAC087/Vitvi08g01841*, which regulates programmed cell death and *WRKY6* expression in Arabidopsis (Chen et al., 2023; Huysmans et al., 2018), and *ERF1* (*Vitvi07g01755*) which forms a complex with WRKY33 to increase the production of defence compounds in Arabidopsis (Zhou et al., 2022) as well as five chitinase genes (Data S11).

### Exogenous β‐1,4‐glucanase/cellulase application increases grafting success

**Figure 7:**
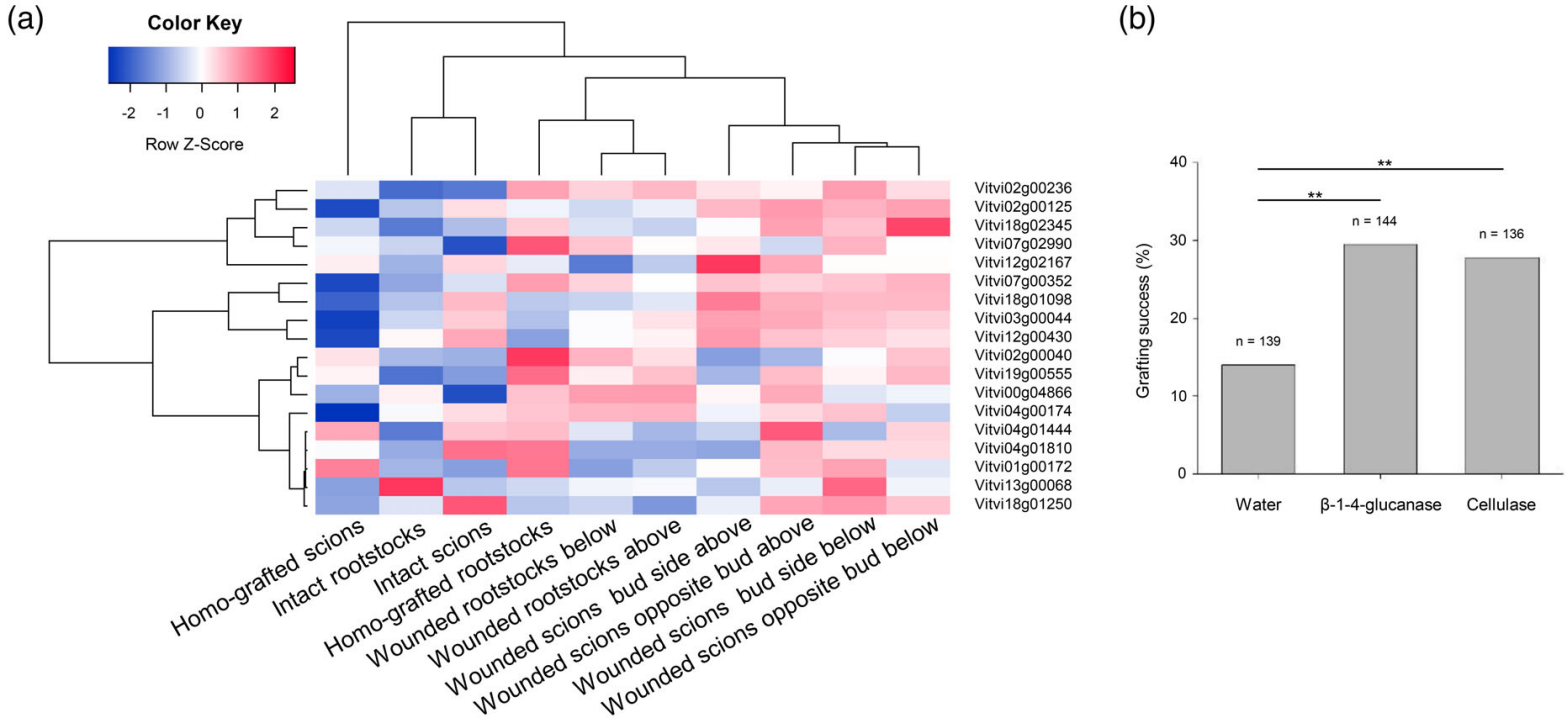
The role of β‐1,4‐glucanases in grapevine graft union formation. (a) Heatmap of the expression of β‐1,4‐glucanases in intact and wounded scions (cuttings with buds) and rootstocks (cuttings without buds) and homo‐grafted grapevine samples taken above and below the wound site/graft interface; (b) grafting success rates after exogenous application of either water, β‐1,4‐glucanases and cellulases to the graft interface, results of a Chi‐squared test are given (**P < 0.01).

There are 25 β‐1,4‐glucanases in grapevine and 18 were expressed in our dataset; the expression of many β‐1,4‐glucanases decreased in response to wounding and/or grafting. However, two genes (*Vitvi02g00236* and *Vitvi19g00555*) up‐regulated after wounding scions in certain cases (Figure 7a). In addition, a *KOR2* orthologue (*Vitvi07g02990*) is in ME11 and another β‐1,4‐glucanase was up‐regulated as early as 3 DAG in a similar experiment (Cookson et al., 2013) (*Vv02s0025g01380* now called *Vitvi02g00125*). We tested whether we could modify grafting success by the exogenous application of β‐1,4‐glucanase and cellulase to the graft interface of a poorly compatible graft combination, *V. vinifera* cv. Ugni blanc onto *V. berlandieri × V. riparia* cv. Rességuier Sélection Birolleau 1 (UB/RSB1 (Loupit et al., 2022)) (Figure 7b). Grafting success rates improved significantly for the β‐1,4‐glucanase and cellulase treatments, reaching 29.5% and 27.8%, respectively, compared to 14% for the water control treatment (Figure 7b).

### Rootstock influences scion transcriptome more than the scion influences the rootstock's transcriptome at 14 DAG

**Figure 8:**
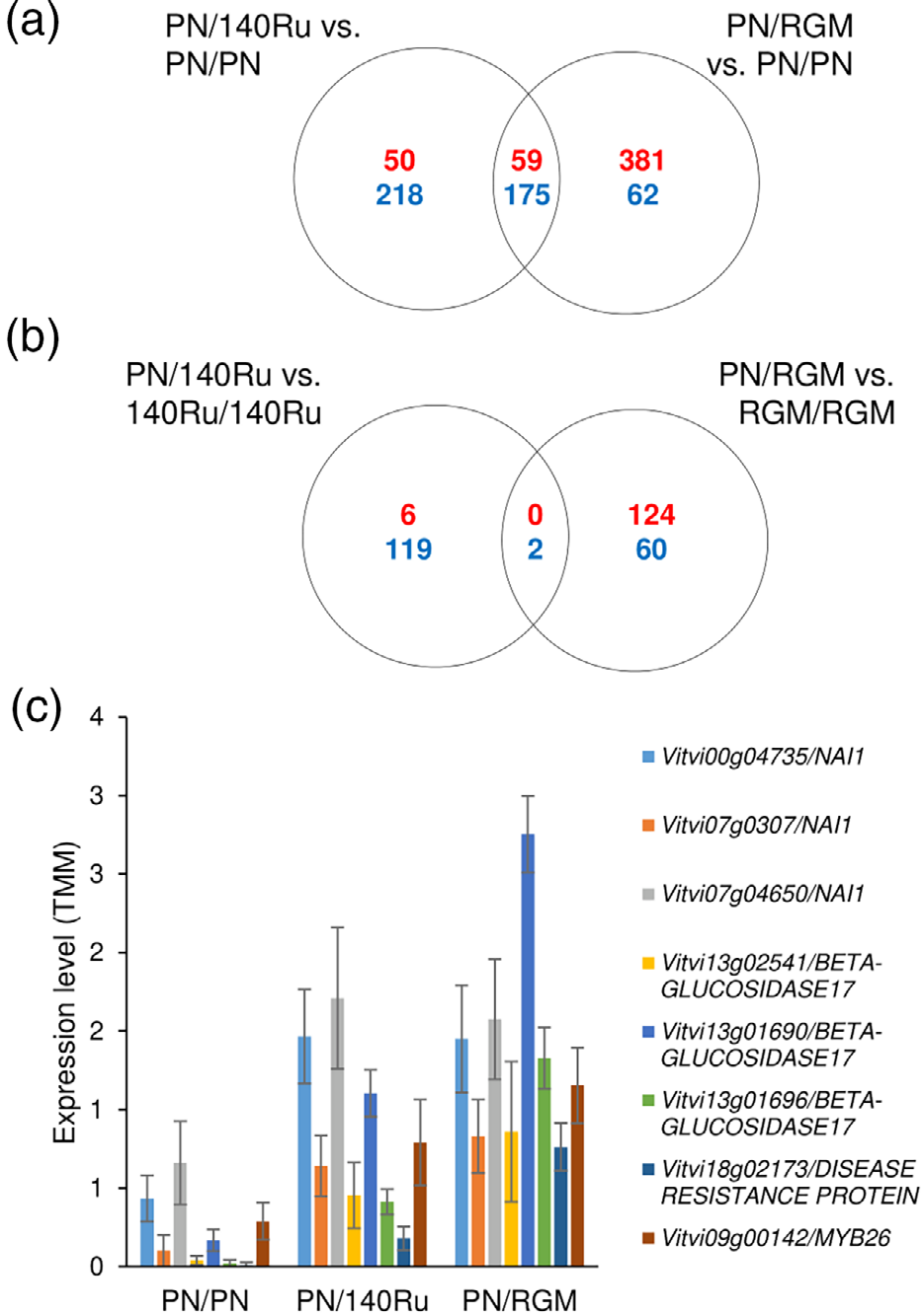
Grafting with a non‐self‐partner modifies gene expression at the graft interface. (a) Venn diagram of the number of genes up‐ (red) and down‐regulated (blue) in the scion in response to grafting with a non‐self‐rootstock. The expression of hetero‐grafts (*V. vinifera* cv. Pinot Noir (PN) scions grafted onto *V. rupestris × V. berlandieri* cv. 140 Ruggeri (140Ru) and *V. riparia* cv. Gloire de Montpellier (RGM) rootstocks) is compared to the homo‐grafted control (PN/PN); (b) Venn diagram of the number of genes up‐ (red) and down‐regulated (blue) in the rootstock in response to grafting with a non‐self‐scion. The expression of hetero‐grafts (PN/140Ru and PN/RGM) is compared to the corresponding homo‐graft control (140Ru/140Ru and RGM/RGM, respectively); (c) expression profiles of the eight key genes more highly expressed in the scion of hetero‐ than homo‐grafts.

Finally, we wanted to characterise the consequences of grafting two different genotypes together by studying the effect a genetically different rootstock can have on the scion transcriptome and metabolome (just above the graft interface) and vice versa (Figure 8a,b, Data S12). Grafting a PN scion with an RGM or 140Ru rootstock triggered the up‐ and down‐regulation of many genes, with 59 up‐ and 175 down‐regulated genes common to both hetero‐graft combinations (Figure 8a, Data S12). The MapMan BINS related to hydrolyases and bHLH transcription factors were enriched in this set of 59 hetero‐graft up‐regulated genes (Table S9). This list of 59 genes included six bHLH transcription factors, including three orthologues of *AT2G22770/NAI1* (*Vitvi00g04735, Vitvi07g03072* and *Vitvi07g04650*) which regulate the formation of endoplasmic reticulum bodies (Matsushima et al., 2003) and three orthologues of *BETA‐GLUCOSIDASE17/AT2G44480* (*Vitvi13g02541*, *Vitvi13g01690* and *Vitvi13g01696*), *DISEASE RESISTANCE PROTEIN/Vitvi18g02173* and an orthologue of *MYB26/Vitvi09g00142*, which is involved in cellulosic secondary wall thickening during pollen development (Figure 8c; Data S12). GO enrichment identified many biological processes from the list of common 175 down‐regulated genes in the scion in response to grafting with a non‐self‐rootstock; the biological processes enriched were synthesis and organisation of the cell wall, including xyloglucans metabolic processes, regulation of transcription, calcium ion transport and regulation of catalytic activity (Table S10). A range of MapMan BINS were enriched in this list of genes down‐regulated in response to grafting with a non‐self‐scion, including external stimuli response (pathogens and cold), phospholipase activity, calcium‐dependent signalling, E3 ligases, AP2/ERF and WRKY transcription factors and solute transport (Table S11). The transcriptome of the rootstock was also modified by grafting with a non‐self‐scion, but few genes responded similarly in both scion/rootstock combinations (Figure 8b). Despite these differences at the transcriptome level, no metabolome changes were observed in the metabolomics analyses (Figure S11).

## DISCUSSION

### Grafting triggers the coordinated up‐regulation of gene expression and accumulation of lipids and phenolic compounds

Grafting and wounding triggered the differential expression of grapevine orthologues of known genes involved in wounding and tissue repair in the model plant Arabidopsis. Here, we identified examples of coordinated regulation between orthologue transcription factors and their target genes. For example, we found that MYB93 may play a role in graft union formation in grapevine, and its expression is up‐regulated at the graft interface and in cut scions of Arabidopsis hypocotyl grafts in comparison to intact tissues (Melnyk et al., 2018). In Arabidopsis, MYB93 regulates lateral root development and the expression of genes related to cell wall synthesis (Uemura et al., 2023). In general, there seems to be some overlap between genes involved in lateral root development and graft union formation (such as *ABERRANT LATERAL ROOT FORMATION4* (Melnyk et al., 2015)). The expression of many genes related to lipid metabolism (such as suberin and jasmonate synthesis) was up‐regulated in response to wounding and grafting (including MYB93, which increases in expression in response to exogenous application of very long chain fatty acids (Uemura et al., 2023) and lipids were accumulated in response to wounding and grafting in our study. Phenolic compounds are often accumulated in response to grafting/wounding in many plant species (Hu et al., 2022; Loupit & Cookson, 2020) including grapevine (Chitarrini et al., 2017; Loupit et al., 2022; Prodhomme et al., 2019). We confirmed this observation and identified the coordinated up‐regulated expression of genes related to secondary metabolite synthesis.

### The transition from dormancy to active growth and grafting shares similar gene expression profiles

During grapevine grafting, we remove canes from cold storage, prepare scions (with buds) and rootstocks (without buds), graft them together and transfer the grafts to a humid callusing room until the callus forms. This change in environmental conditions mimics those of spring‐induced dormancy release and activation of growth (such as bud burst and adventitious root development). Here, we show that the transition from dormancy to active growth triggers the DE of thousands of genes in the wood, in particular those related to the regulation of cell cycle progression and cell wall biogenesis, which could be linked to the activation of cambial activity. A similar transcriptome study has been done on the gene expression changes occurring in grapevine wood during bud burst and similar results were obtained (Noronha et al., 2021). We found considerable overlap between the transcriptome remodelling occurring in response to grafting and the transition from dormancy to active growth, which corroborates our previous study (Cookson et al., 2013). However, we observed few changes in the metabolite profile of the wood in the same samples from 0 to 14 DAG; this could be genotype‐specific differences. This could be due to the fact that metabolite changes only occurred in a few cell types and were largely diluted in the tissue samples analysed (whereas RNA extractions probably contains a disproportionate amount of RNA from a small proportion of highly metabolically active, RNA‐rich tissues). However, discordance between metabolomics and transcriptomics data is frequently observed in plant science and could be due to a variety of factors such as post‐transcriptional and post‐translational modifications.

The wood transcriptome response to the transition from dormancy to active growth differed between intact un‐grafted scions and rootstocks (i.e. between cuttings with and without buds). In particular, many genes related to cell wall synthesis were more highly expressed in scions than in rootstocks, suggesting that spring activation of metabolism and growth may be enhanced by the presence of auxin originating from the bud.

### Grafting and wounding induce asymmetric gene expression responses above and below the graft/wound site

Following grafting or wounding, many genes show an asymmetric expression across the graft interface/wound site in plants. In addition, we found that more genes were asymmetrically expressed in response to grafting than wounding, presumably because communication is maintained through the intact tissues of wounded stems. We also tested whether the presence and location of the bud relative to the wound site also affected the transcriptome response. Scions with a wound site below the bud showed more pronounced asymmetric gene expression responses than those wounded on the opposite side of the bud, suggesting that axial movement of signals from developing buds (such as auxin) drives this asymmetric response and that axial communication is more efficient than abaxial communication in grapevine.

Melnyk et al. (2018) found that the proportion of genes asymmetrically expressed across the graft interface reduced over time in Arabidopsis hypocotyl grafts and that it was partially due to differences in carbon status and auxin concentration across the graft interface. We observed a similar overlap between genes DE above and below the graft interface and carbon starvation and the presence of a bud in grapevine. In addition, genes related to the functional categories carbohydrate metabolism and auxin signalling were asymmetrically expressed across the graft interface (with higher expression above than below the interface); similar results have been found in other studies on grafting herbaceous grafts of annuals (Melnyk et al., 2018; Xie et al., 2019) and nut trees (Mo et al., 2024). The asymmetric expression of genes related to carbohydrate metabolism in grapevine grafts does seem surprising, as presumably, carbohydrates are not limiting in woody grafts of perennial crops. This could suggest that signals coming from the bud interact with the regulation of carbohydrate metabolism in the wood. The comparison between genes asymmetrically expressed across the graft interface with genes responsive to carbon starvation in suspension cells (Berger et al., 2024) indicates that carbon starvation in the rootstock possibly drives the up‐regulated expression of many genes in the rootstock relative to the scion. The asymmetric transcriptome response to grafting was accompanied by an asymmetric accumulation of some metabolites across the graft interface, which has not previously been reported. On the other hand, the asymmetric expression of genes related to auxin signalling across the graft interface in grapevine is expected due to the presence of a bud in the scion.

From our experimental design, we were able to characterise the differences in wounding responses between scions and rootstocks, and how this relates to the asymmetry of gene expression responses to grafting. It appears that some of the asymmetry of gene expression across the graft interface is due to the different responses of scions and rootstock to wounding, in particular for those genes specifically up‐regulated in the rootstock that overlapped with wound‐induced genes specific to un‐grafted rootstocks.

### There are grafting‐ and wounding‐specific gene expression responses

Although wounding and grafting responses are very similar, some grafting and wounding‐specific responses were identified at the transcriptome level. Notably, the genes that are involved specifically in the response to wounding included gravity signalling proteins, which in Arabidopsis regulate auxin distribution in response to gravity. The expression of these proteins could be related to the re‐direction of auxin around the wounded tissue (Asahina et al., 2011). The graft‐specific genes included genes related to chitin catabolic processes; chitin‐related genes are often up‐regulated in response to grafting (Melnyk et al., 2018). Plant chitinases are known to have roles in plant defence against pathogens; they also have roles in plant responses to abiotic stresses and cell wall development (Grover, 2012). The graft‐specific genes included various transcription factors involved in defence responses, such as grapevine orthologues of *WRKY9*, *WRKY6*, *WRKY33*, *ANAC087* and *ERF1*.

### β‐1,4‐glucanases are involved in grafting in grapevine

Certain scion/rootstock combinations of grapevine have poor grafting success rates (Tedesco et al., 2022), in particular UB/RSB1 (Loupit et al., 2022). We found that exogenous application of β‐1,4‐glucanase/cellulase increased grafting success in this scion/rootstock combination, suggesting that a role of β‐1,4‐glucanases in graft union formation is conserved across many species such as tobacco (Notaguchi et al., 2020), Arabidopsis (Notaguchi et al., 2020), oriental melon (Zhu et al., 2024) and grapevine. This also supports the idea that the genes DE during grafting have a role in graft union formation.

### The rootstock modifies the scion transcriptome

There have been numerous studies comparing the genes differentially expressed between compatible and incompatible grafts (Assunção et al., 2019; Chen et al., 2017; Febres et al., 2024; Thomas et al., 2021), different compatible hetero‐grafts (Cookson et al., 2014; Mo et al., 2023), and hetero‐ and homo‐grafts (Cookson et al., 2014; Ji et al., 2022). In general, these studies have been done on bulk graft interface samples and often without appropriate controls (as reviewed by Loupit and Cookson (2020)). Here we analysed the scion and rootstock tissues separately, and we showed that grafting with a non‐self‐rootstock modifies scion gene expression just above the graft interface (similar to a study done on Ziziphus species (Zhang et al., 2024)). We found that hetero‐grafting with a non‐self‐rootstock particularly increased the expression of genes related to the formation of ER bodies, orthologues of *NAI1*, which regulates their formation, and *BETA‐GLUCOSIDASES* (beta‐glucosidases are the main component of ER bodies) (Matsushima et al., 2003). Endoplasmic reticulum bodies are formed in response to wounding and presumably have roles in plant defence (Matsushima et al., 2003); it is possible that grafting with a non‐self‐rootstock triggers a stress response that will induce the formation of ER bodies in the scion. In addition to these bHLH transcription factors, the expression of other transcription factors and potential regulatory genes was up‐regulated in the scion of hetero‐grafts relative to homo‐graft controls. In contrast to the study of Ziziphus species by Zhang et al. (2024), we did not see a significant up‐regulated expression of genes related to primary and secondary metabolism, and no differences in metabolites were observed; this could be because we grafted dormant plant material and sampled at an early time point after grafting.

### Conclusion

Our work explored the different factors regulating gene expression and metabolite accumulation during graft union formation in a woody, perennial crop. We examined gene expression in response to grafting, wounding, spring activation of growth, the presence and position of a bud on the stem, the asymmetry of responses of the scion and rootstock and the genotype grafted. We showed that spring‐induced dormancy release has a huge impact on gene expression, highlighting the complex mechanisms occurring in parallel during the grafting of perennial plants. We compared graft‐ and wound‐responsive genes and showed that cellulase/β‐1‐4 glucanase activities are involved in grafting, and that exogenous application of these enzymes increases grafting success in grapevine. Finally, we showed that the strong influence of the rootstock on the scion transcriptome indicates that at 14 DAG, even before the callus is formed and the vascular vessels are generated, the expression of genes in the scion is challenged in response to a non‐self‐rootstock. In the future, this dataset could complement quantitative genetics studies of grafting success and aid in the selection of causal genes underlying quantitative trait loci.

## MATERIALS AND METHODS

### Plant material, grafting procedure and sampling

Grafts and un‐grafted scion and rootstock cuttings were made in early spring 2021; *V. vinifera* cv. Pinot Noir (PN) and *V. rupestris × V. berlandieri* cv. 140 Ruggeri (140Ru) were from La Chambre de l'Agriculture de l'Aude (Carcassonne, France) and *V. riparia* cv. Gloire de Montpellier (RGM) was from Mercier (Vix, France) (Table S12). The grafting procedure used was the same as the one described in Loupit, Fonayet, et al. (2023). Briefly, dormant stems were made into one‐bud scions and de‐budded rootstocks, and then grafted together with an omega‐shaped blade using a grafting machine (Omega Star, Chauvin, France). The wounds were made using the same machine, but only cut until the middle of the stem. All intact, wounded and grafted material was dipped in melted wax (Staehler Rebwaches pro with 0.0035% dichlorobenzoic acid) and then put in plastic boxes and transferred to a callusing room of high humidity and at 28°C. Dormant woody cuttings just before grafting were sampled from the three genotypes (0 DAG) and the different graft combinations, and intact and wounded un‐grafted scions and rootstocks were sampled at 14 DAG just before the start of callus proliferation at the graft interface (Figure 1). For this, four pools of five grafts/cuttings were randomly sampled per experimental modality. Approximately 0.5 cm of tissue was taken from above or below the graft interface or wound site, and immediately immersed in liquid nitrogen and stored in the freezer at −80°C. For the cuttings without wounds, 1 cm of wood was sampled at the same location as the graft interface or wound site. The metadata describing these samples is given in Data S1.

In early spring 2022, we tested whether exogenous application of β‐1,4‐glucanase (5 U/mL) and cellulase (500 U/mL) modifies grafting success of a poorly compatible scion/rootstock combination, *V. vinifera* cv. Ugni blanc (UB) grafted onto *V. berlandieri × V. riparia* cv. Rességuier Sélection Birolleau 1 (RSB1) (wood from Mercier, Vix, France). After cutting the scion and rootstock, the cut surfaces were immersed in a solution containing either water, β‐1,4‐glucanase (β‐1,4‐glucanase from *Trichoderma longibrachiatum*, Sigma‐Aldrich) or cellulase (cellulase Onozuka R‐10, Duchefa) for 5 s before assembling the graft. Then, the same grafting procedure as Loupit, Fonayet, et al. (2023) was followed. After callus proliferation, grafts were planted in a field nursery covered with a black plastic and uprooted in January 2023. Grafting success was assessed by manually testing the mechanical resistance of the graft to breaking under force; this technique is typically used to assess whether grafts are suitable for commercialisation by grapevine nurseries (Carrere et al., 2022). The percentage of grafting success corresponds to the ratio of grafts that passed this test without breaking to the total number of tested grafts.

### Analysis by UHPLC‐QqQ

The chemicals, standards and extraction protocol used are the same as given in Loupit, Fonayet, et al. (2023). The analysis protocol was the same as described by Loupit et al. (2020) with some modifications (Table S13) and a different column (Agilent ZORBAX RRHD SB‐C18 [2.1 mm × 100 mm, 1.8 μm]).

### Untargeted metabolome analysis

Semi‐polar compounds were extracted using automated high‐throughput ethanol extraction procedures at the MetaboHUB‐Bordeaux Metabolome (https://metabolome.u‐bordeaux.fr/) from 35 mg of fresh ground material, following previously established protocols (Luna et al., 2020). All samples were randomised and injected alternately with extraction blanks (prepared without plant material and used to rule out potential contaminants detected by untargeted metabolomics) and 13 quality control samples that were prepared by mixing 10 μL from each sample. Quality control samples were injected every eight runs and used for the correction of signal drift during the analytical batch and the calculation of coefficients of variation for each metabolomic feature, so only the most robust ones are retained for chemometrics (Broadhurst et al., 2018).

Untargeted analysis was performed on a UHPLC Vanquish (Thermo Fisher Scientific) coupled to a Q‐Exactive Plus mass spectrometer (Thermo Fisher Scientific). One microlitre of sample was injected on a Phenomenex Luna^®^ Omega Polar C18 column (50 × 2.1 mm, 1.6 μm) at 40°C, and a gradient of solvent A (milliQ water—0.1% formic acid) and solvent B (acetonitrile—0.1% formic acid) with a flow of 0.5 mL/min^−1^ was used. The gradient elution was set as follows: 0–11.5 min: 1%–40% solvent B; 11.5–12.5 min: 40%–95% solvent B; 12.5–14 min: 95% solvent B; 14.5–16 min: 1% solvent B.

The mass spectrometry data was acquired in negative polarity at 140.000 FWHM resolution with an automatic gain target at 3e^6^ and maximum IT of 100 ms. The source conditions were as follows: Spray voltage: 3000 V; Sheath gas: 45 a.u; Auxiliary gas: 15 a.u; Capillary temperature: 320°C; Probe heater temperature: 250°C; S‐lens RF level: 100. The experiments were in full scan (mass range: 70–1050 m/z)—data‐dependent MS2 with top three precursors and normalized collision energies of 15, 30 and 45 using a dynamic exclusion of 5 s.

Raw data were processed using MS‐DIAL v4.9 (Tsugawa et al., 2015), yielding 17 047 RT‐m/z features, following optimised parameters detailed in Methods S1. We also implemented retention time correction in MS‐DIAL, and the metabolomic signals were normalised according to the LOWESS method based on the QC samples. After data curation (blank check, SN > 10, CV QC < 30%), we retained 4749 final features (Data S4), of which 152 matched with MS1 and MS2, 2520 suggested features matched with MS1 only and 2077 unknowns (no match). Annotations were performed based on MS1 spectra and MS2 DDA fragmentation information using the FragHUB database, including thousands of natural products (Dablanc et al., 2024). Thus, putative annotation of differentially accumulated metabolites resulted from MS‐DIAL screening of the MS1 detected exact HR m/z and MS2 fragmentation patterns (Tsugawa et al., 2015). Additionally, the InChiKeys of annotated features were employed within ClassyFire to automatically generate a structural ontology for chemical entities (Djoumbou Feunang et al., 2016).

### RNAseq analysis

Total RNA was extracted using Spectrum Plant Total RNA Kit (Sigma‐Aldrich) with some modifications. A total of 80–100 mg of material was extracted with 900 μL of lysis solution with 40 mg of polyvinylpolypyrrolidone. The resulting solution was extracted once with chloroform:isoamyl alcohol (24:1, v:v) and then RNA was extracted according to the manufacturer's recommendations. RNA samples were sent to BGI platform (Shenzen, China) for sequencing on DNBseq platform where 100 cDNA libraries corresponding to four biological replicates for each experimental modality were generated. At least around 115–128 million sequencing reads were generated for each sample.

For bioinformatic analysis, the nf‐core/rnaseq pipeline (Ewels et al., 2020) was used to generate raw gene counts. Raw RNA‐seq reads were filtered and mapped to the *V. vinifera* genome (PN40024.v4, (Velt et al., 2023). For biostatistic analysis, edgeR (version 3.42.4), a Bioconductor package (with software R version 4.0.4 (R Core Team, 2021) and Rstudio version 1.2.5019 (RStudio Team, 2020) was used for normalisation and differential expression analysis (Robinson et al., 2010). Libraries were normalised by the trimmed mean of M values (TMM) normalisation. The sample clustering and principal component analysis are shown in Figures S12 and S13. A design matrix was built with a replicate effect before differential expression analysis with GLM tests for a condition used as a reference. In all other cases, for simple comparison between two conditions, a pairwise exact test was executed. Genes were considered differentially expressed (DE) only when the absolute log₂‐fold change was higher than 1.5 and the adjusted *P*‐value (FDR) < 0.05. In order to identify common DE genes with different comparisons, Venn diagrams were made by VENNY 2.1 (Oliveros, 2007–2015).

Gene annotations were obtained from the Grapedia website (https://grapedia.org/, [Navarro‐Payá et al., 2022]) and the best Arabidopsis hit and gene description were obtained from the TAIR website (https://www.arabidopsis.org/ (Rhee et al., 2003)).

Gene ontology (GO) terms were obtained from the Grapedia website (https://grapedia.org/) and GO term enrichment analysis was done with the topGO R package using Fisher's exact test with a significance level α ≤ 0.05 (Alexa & Rahnenfuhrer, 2024).

Mapman BINS were assigned using Mercator 4v5 on the *V. vinifera* genome (PN40024.v4) (Schwacke et al., 2019). Enrichment of MapMan BINS was tested for significance using the 38 516 genes in the mapping file as a reference with a Fisher's exact test with Bonferroni correction with a significance level α ≤ 0.05.

### Co‐expression network analysis

RNAseq count data obtained from the R package edgeR after TMM normalisation was used to construct gene co‐expression signed networks using the R package WGCNA (Langfelder & Horvath, 2008) after log₂ (normalised count +1) transformation. We used a soft threshold of 17 that resulted in a more than 80% model fit to scale‐free topology. Then gene networks were computed with co‐expression relationships (bi weight mid correlation coefficients (bicor)) as signed networks (maxBlockSize = 15 000, minModuleSize = 150, deepSplit = 3). Each module was described by ME (Data S2) and scaled intra‐modular connectivity was computed for all genes (Data S3).

Sparse Partial Least Squares (sPLS) approach was used to investigate relationships between WGCNA module eigengenes and untargeted metabolite features using the R package mixOmics (version 6.24.0 (González et al., 2012; Le Cao et al., 2016; Lê Cao et al., 2009; Rohart et al., 2017)). A regression model was applied on ME1–30 and on the 100 best metabolite features with LASSO penalisation, for each component (the *Q* ^2^_total_ was over 0.0975 for the first two components, Figure S2).

### Statistics

Principal component analysis (PCA), HeatMaps and ANOVA test were done on RStudio (version 1.2.5019 [RStudio Team, 2020]) with R software (version 4.0.4, (R Core Team, 2021) using the packages ggplot2 (Wickham, 2016), gplot (Warnes et al., 2009), FactoMineR (Lê et al., 2008), factoextra (Kassambara & Mundt, 2020) and agricolae (de Mendiburu & Yaseen, 2023). 3D‐PCA, PLS‐DA, volcano plot and boxplots for WGCNA modules were done using MetaboAnalyst (version 6.0) (Pang et al., 2024). The effect of exogenous application of β‐glucanases or cellulases on UB/RSB1 grafting success was tested with a Chi‐squared test using R software. Some graphs were also drawn with SigmaPlot version 16.

## Supporting information


**Figure S1.** Boxplots of eigengene values for ME24, 9, 11, 17 and 28.

**Figure S2.** Expression of key genes in ME11 in intact, wounded and grafted scions and rootstocks.

**Figure S3.** Heatmap of module‐metabolite.

**Figure S4.** Tuning the number of sPLS components on module eigengene and untargeted metabolic data.

**Figure S5.** The concentration of resveratrol and the expression of a stilbene synthase in intact, wounded and grafted scions and rootstocks.

**Figure S6.** Genes differentially expressed and metabolites differentially accumulated in the wood during spring‐induced dormancy release.

**Figure S7.** Relationship between the genes differentially expressed in wood tissue during the transition from dormancy to active growth and those differentially expressed in response to grafting.

**Figure S8.** Principle component (PC) analysis of the metabolite features in intact and wounded rootstocks and scions.

**Figure S9.** Genes differentially expressed and metabolites differentially accumulated above and below the graft interface of grapevine homo‐grafts.

**Figure S10.** Venn diagrams of the genes differentially expressed between the scion and rootstock of homo‐grafts compared with (A) the genes responding to carbon (C) starvation (Berger et al., 2024), (B) the genes responding to the presence of a bud (i.e. differentially expressed between intact scions and rootstocks), the genes (C) up‐regulated and (D) down‐regulated by wounding un‐grafted scions and rootstocks.

**Figure S11.** Principle component (PC) analysis of the metabolite features present above and below the graft interface of homo‐ and hetero‐grafts of grapevine 14 days after grafting.

**Figure S12.** Sample correlation and clustering heatmap.

**Figure S13.** Principle component (PC) analysis of samples.

**Data S1.** Sample MetaData and BioSamples identifiers.

**Data S2.** WGCNA module eigengene values for each sample.

**Data S3.** WGCNA module assignation and gene connectivity.

**Data S4.** Relative content for the 4749 features extracted from untargeted metabolic analysis and the list of features extracted from sPLS model for component 2.

**Data S5.** Concentration of the 39 secondary metabolites quantified by HPLC‐QqQ.

**Data S6.** Transcriptome expression matrix (29 983 genes × 100 samples), gene counts normalised by trimmed mean of M values (TMM) from RNAseq data with histogram visualisation.

**Data S7.** Genes differentially expressed between dormant stems stored at 4°C and after transfer to warm forcing conditions for 14 days.

**Data S8.** Genes differentially expressed between un‐grafted scions and rootstocks of grapevine.

**Data S9.** Genes differentially expressed in response to wounding in scions and rootstocks relative to intact scions and rootstocks, respectively.

**Data S10.** Genes asymmetrically expressed across the graft and wound interface of *Vitis vinifera* cv. Pinot noir 14 days after wounding/grafting.

**Data S11.** Genes differentially expressed between grafted and wounded scions and rootstocks of *Vitis vinifera* cv. Pinot Noir 14 days after grafting/wounding.

**Data S12.** Genes differentially expressed in the scion between hetero‐ and homo‐grafts of grapevine 14 days after grafting.

**Data S13.** General statistics describing RNA sequencing data.

**Table S1.** MapMan BINs enriched in the genes presented in ME17.
**Table S2.** Gene Ontology (GO) terms enriched in the genes present in ME17.
**Table S3.** Gene Ontology (GO) terms enriched in the genes present in ME11.
**Table S4.** MapMan BINs enriched in the genes presented in ME11.
**Table S5.** MapMan BINs enriched in the 848 genes more highly expressed in the rootstock than scion 14 days after grafting.
**Table S6.** MapMan BINs enriched in the 594 genes more highly expressed in the scion than rootstock 14 days after grafting.
**Table S7.** MapMan BINs enriched in the 364 genes more highly expressed in response to wounding than grafting in both scions and rootstocks.
**Table S8.** MapMan BINs enriched in the 477 genes more highly expressed in response to grafting than wounding in both scions and rootstocks.
**Table S9.** MapMan BINs enriched in the 59 genes more highly expressed in scions of homo‐ than hetero‐grafts.
**Table S10.** Gene Ontology (GO) terms enriched in the 175 genes more highly expressed in scions of homo‐ than hetero‐grafts.
**Table S11.** MapMan BINs enriched in the 175 genes more highly expressed in scions of homo‐ than hetero‐grafts.
**Table S12.** Grafts and cuttings used in this study.
**Table S13.** Parameters for the analysis of metabolites by HPLC‐QqQ in MRM mode.

**Data S14.** **Methods S1.** Optimised parameters for raw data processing of untargeted metabolomics analysis.

## Data Availability

All raw data are publicly available, RNAseq data are available at https://www.ebi.ac.uk/biostudies/arrayexpress/studies/E‐MTAB‐14101, and untargeted and targeted metabolomic data at https://doi.org/10.57745/GJRUQG and https://doi.org/10.57745/GCUYCF, respectively.
