# Supplementary figures and images for: Graft union formation involves interactions among bud signals, carbon availability, dormancy release, wound responses and non‐self‐communication in grapevine
Source: Plant J. 2025 Jun 11;122(5):e70244. doi: 10.1111/tpj.70244 (PMC12155988; doi:10.1111/tpj.70244)

**A**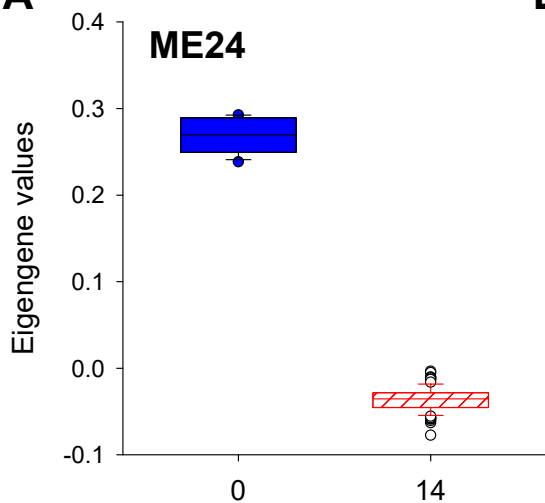**B**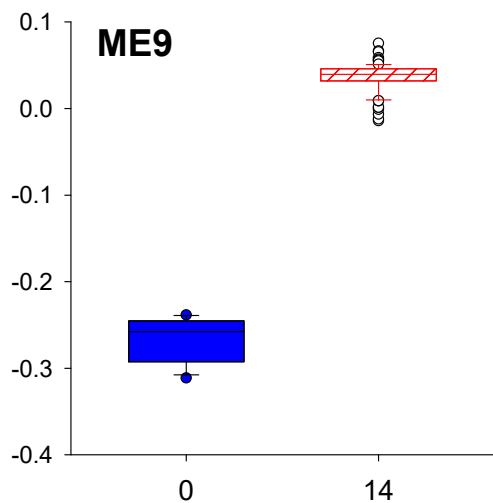**C**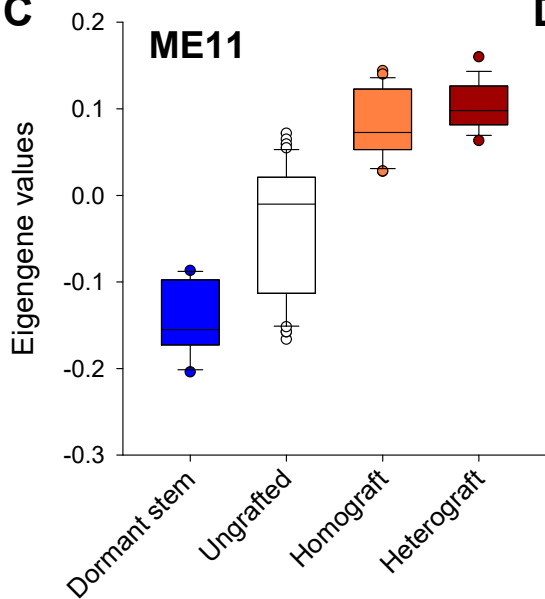**D**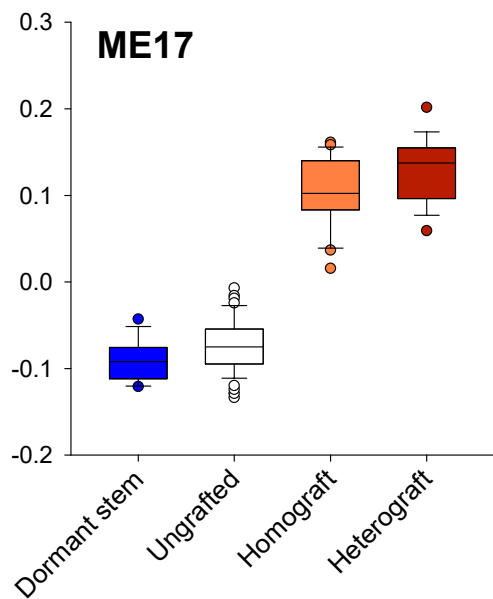**E**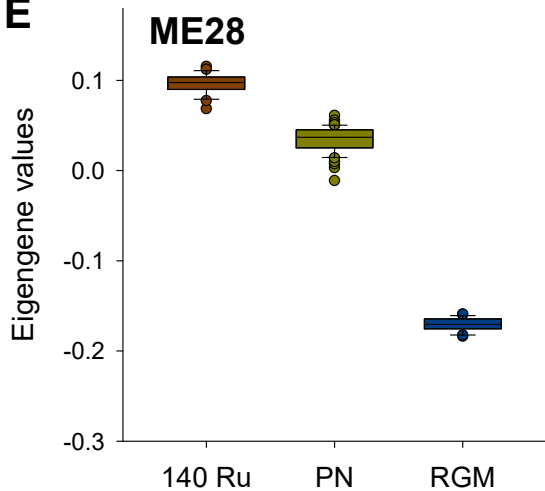

Supplement: Supplementary file 1 — Figure S1. Boxplots of eigengene values for ME24, 9, 11, 17 and 28. [file TPJ-122-0-s019.pdf]

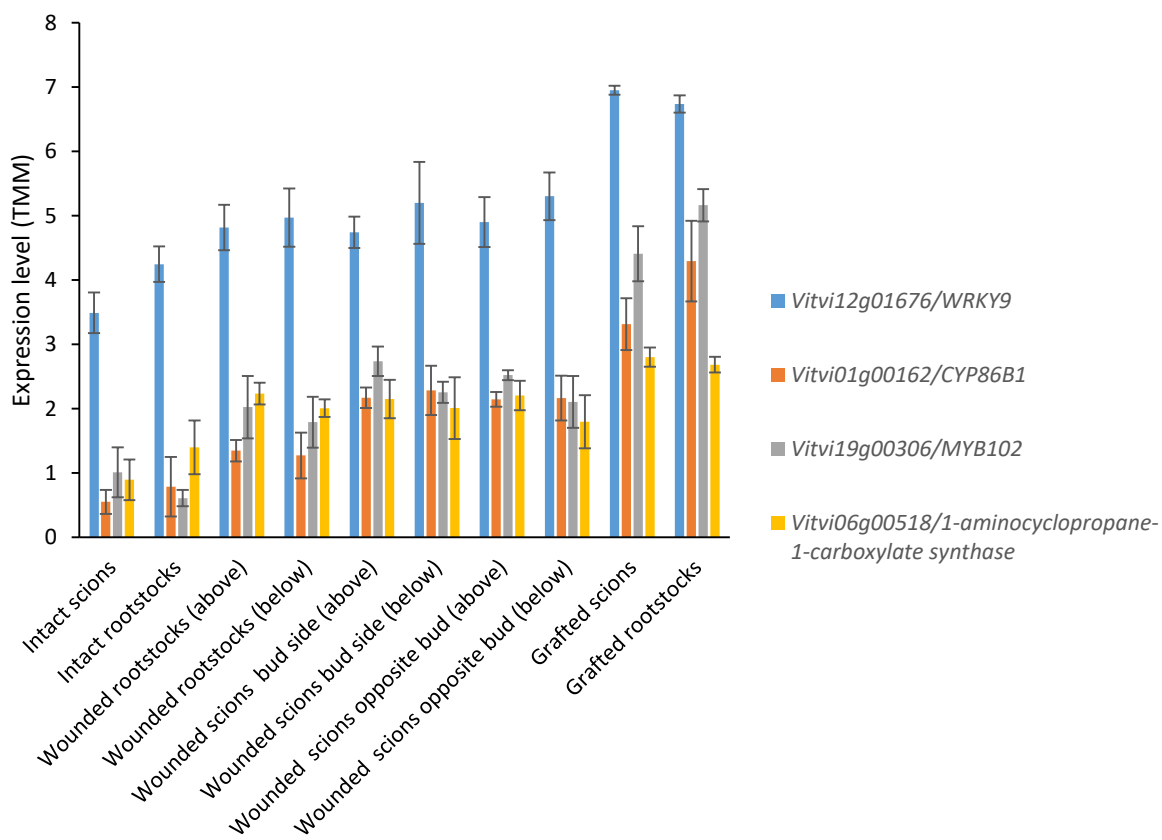

Supplement: Supplementary file 2 — Figure S2. Expression of key genes in ME11 in intact, wounded and grafted scions and rootstocks. [file TPJ-122-0-s027.pdf]

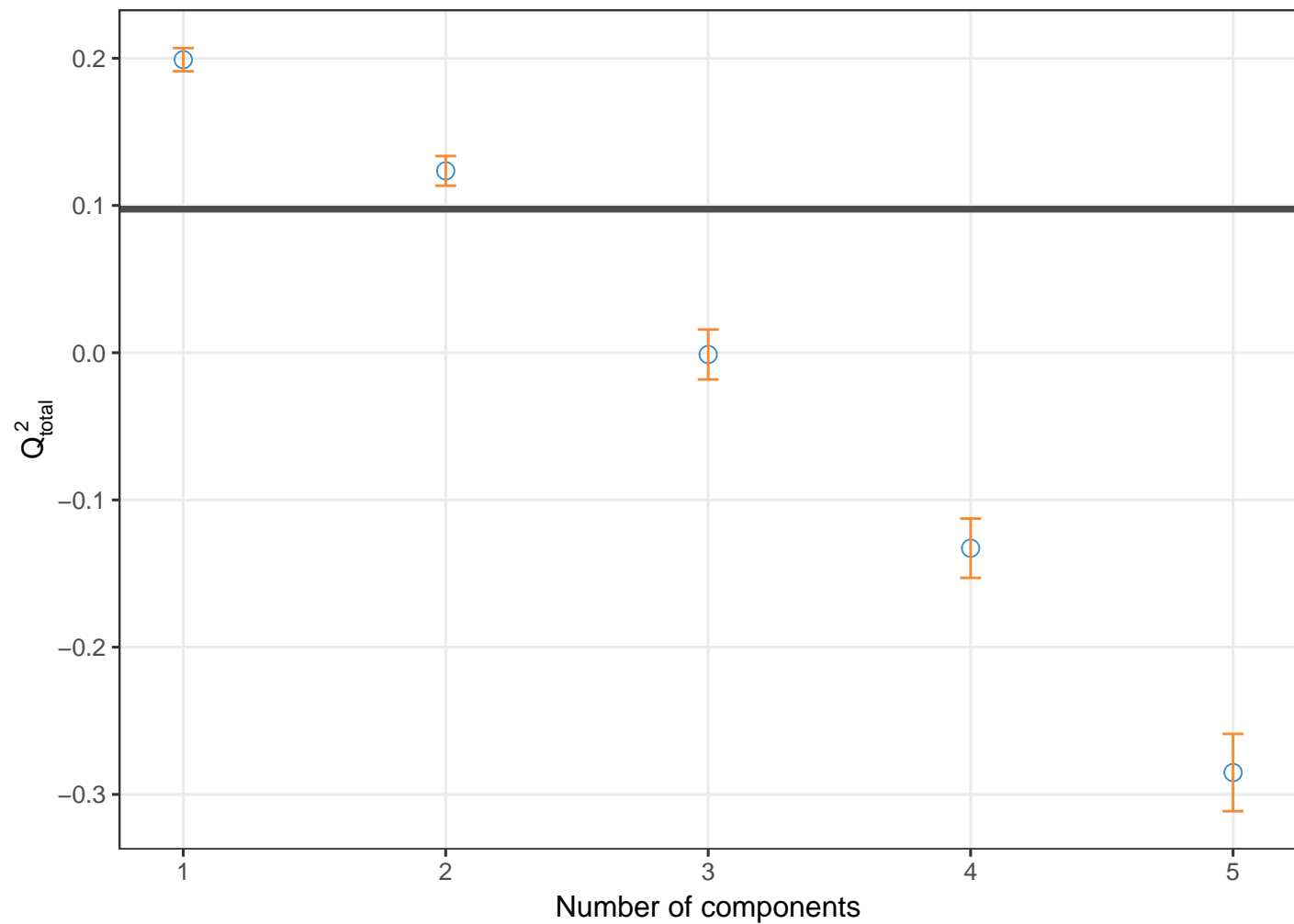

Supplement: Supplementary file 4 — Figure S4. Tuning the number of sPLS components on module eigengene and untargeted metabolic data. [file TPJ-122-0-s015.pdf]

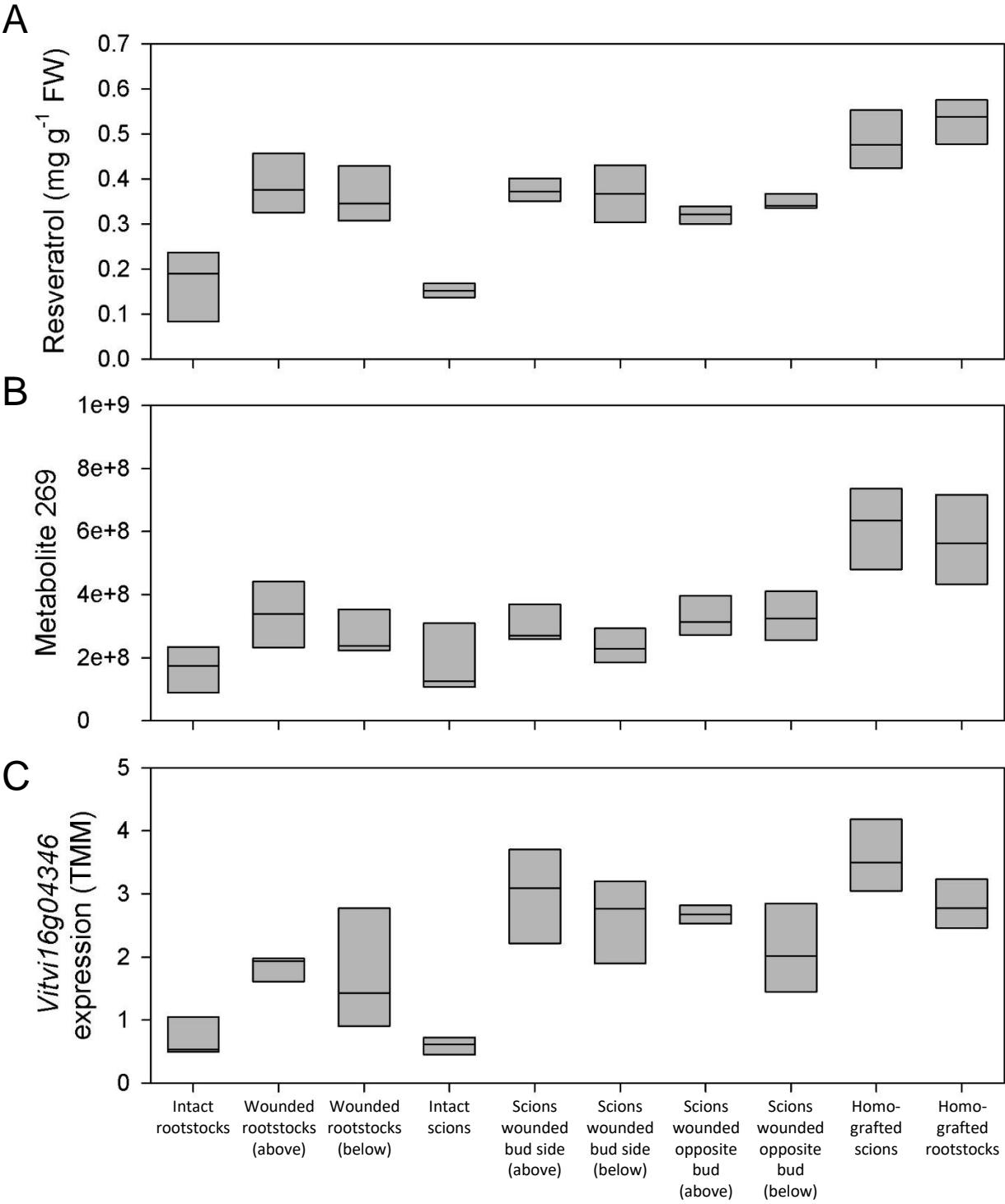

Supplement: Supplementary file 5 — Figure S5. The concentration of resveratrol and the expression of a stilbene synthase in intact, wounded and grafted scions and rootstocks. [file TPJ-122-0-s006.pdf]

A

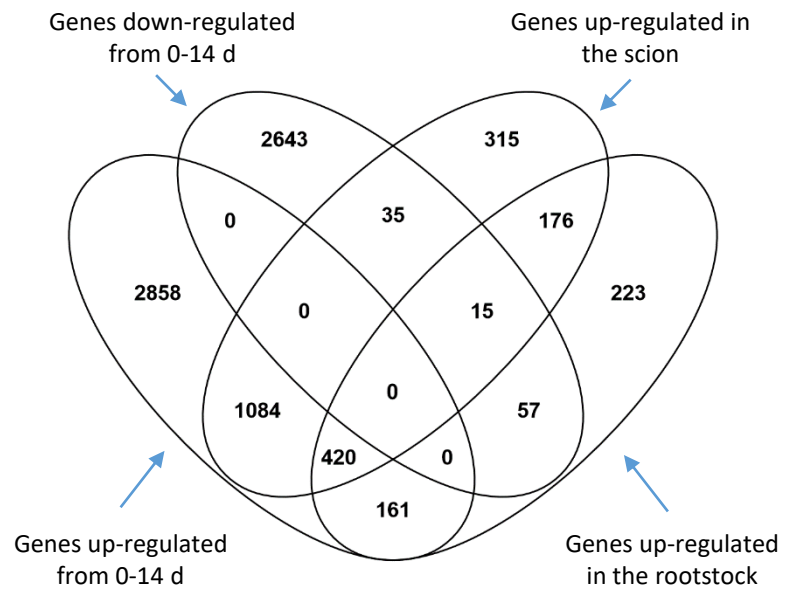

B

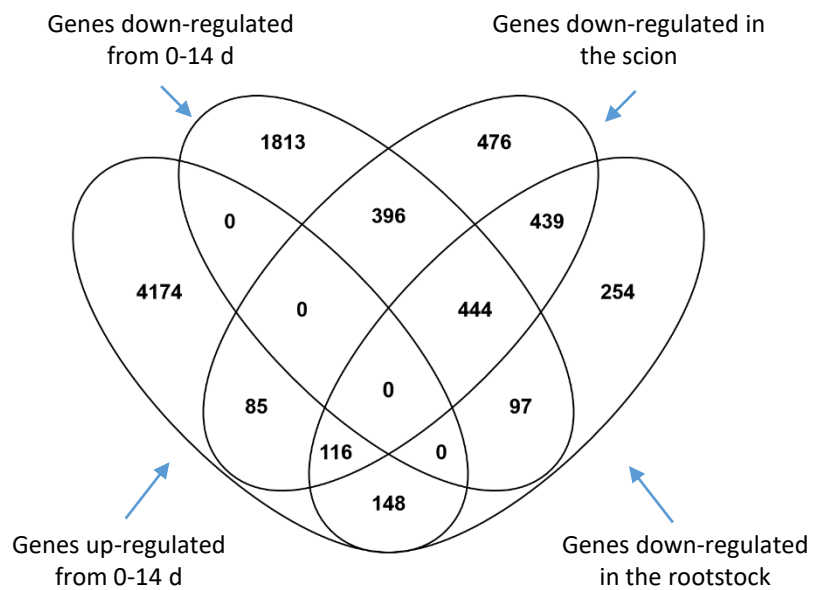

Supplement: Supplementary file 7 — Figure S7. Relationship between the genes differentially expressed in wood tissue during the transition from dormancy to active growth and those differentially expressed in response to grafting. [file TPJ-122-0-s017.pdf]

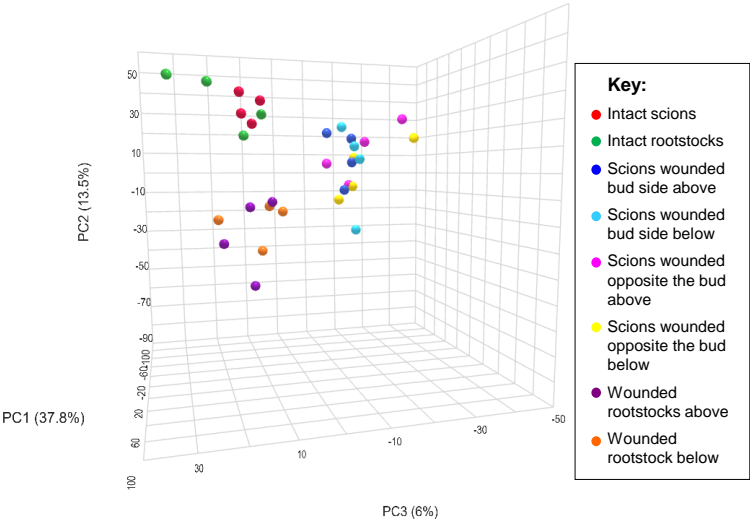

Supplement: Supplementary file 8 — Figure S8. Principle component (PC) analysis of the metabolite features in intact and wounded rootstocks and scions. [file TPJ-122-0-s010.pdf]

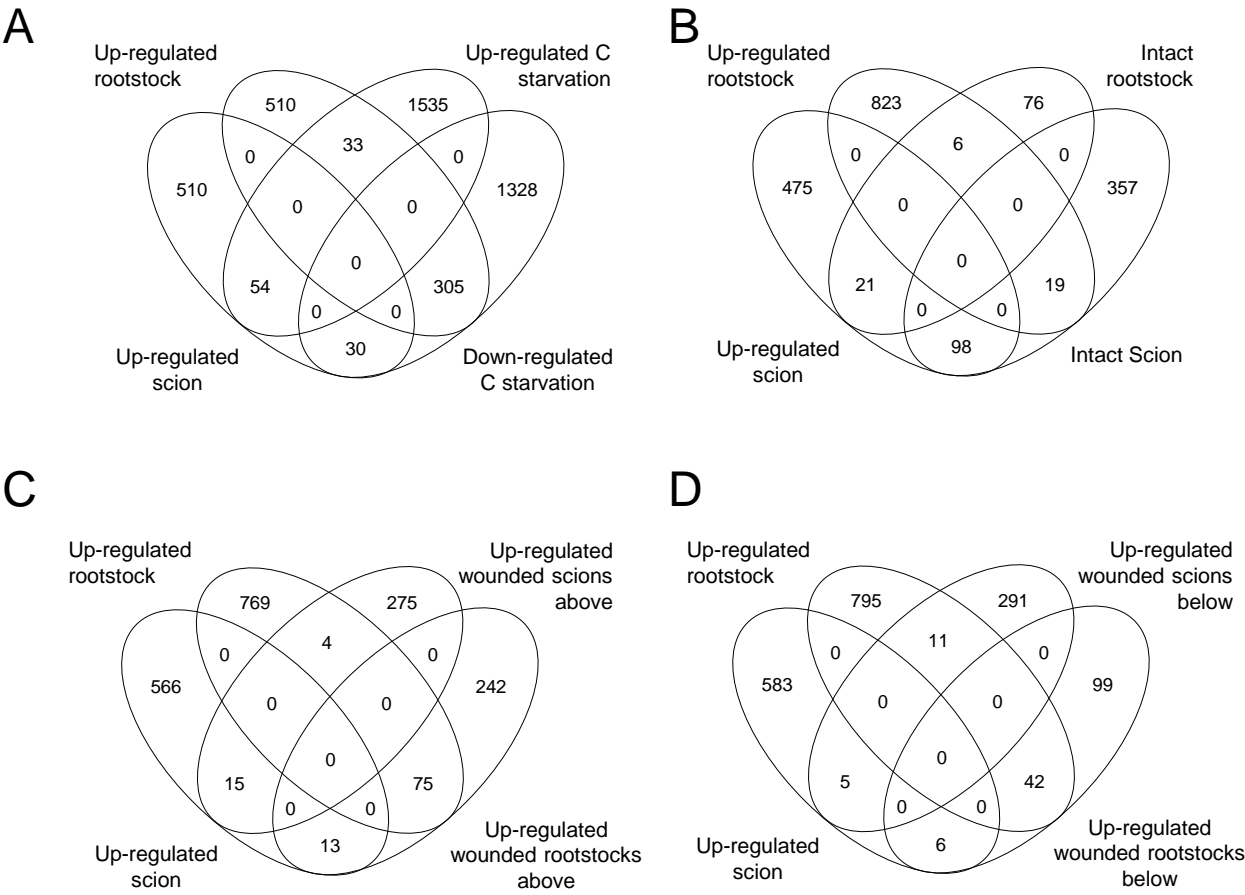

Supplement: Supplementary file 10 — Figure S10. Venn diagrams of the genes differentially expressed between the scion and rootstock of homo‐grafts compared with (A) the genes responding to carbon (C) starvation (Berger et al., 2024), (B) the genes responding to the presence of a bud (i.e. differentially expressed between intact scions and rootstocks), the genes (C) up‐regulated and (D) down‐regulated by wounding un‐grafted scions and rootstocks. [file TPJ-122-0-s004.pdf]

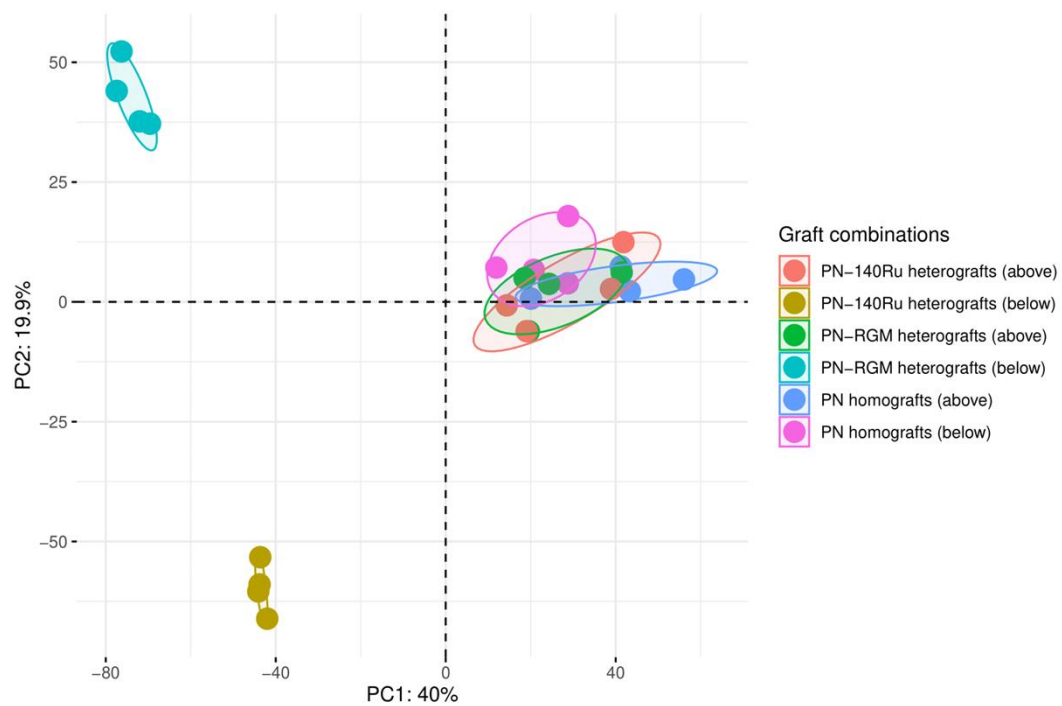

Supplement: Supplementary file 11 — Figure S11. Principle component (PC) analysis of the metabolite features present above and below the graft interface of homo‐ and hetero‐grafts of grapevine 14 days after grafting. [file TPJ-122-0-s022.pdf]

# PlotIndiv

PC2: 21% expl. var

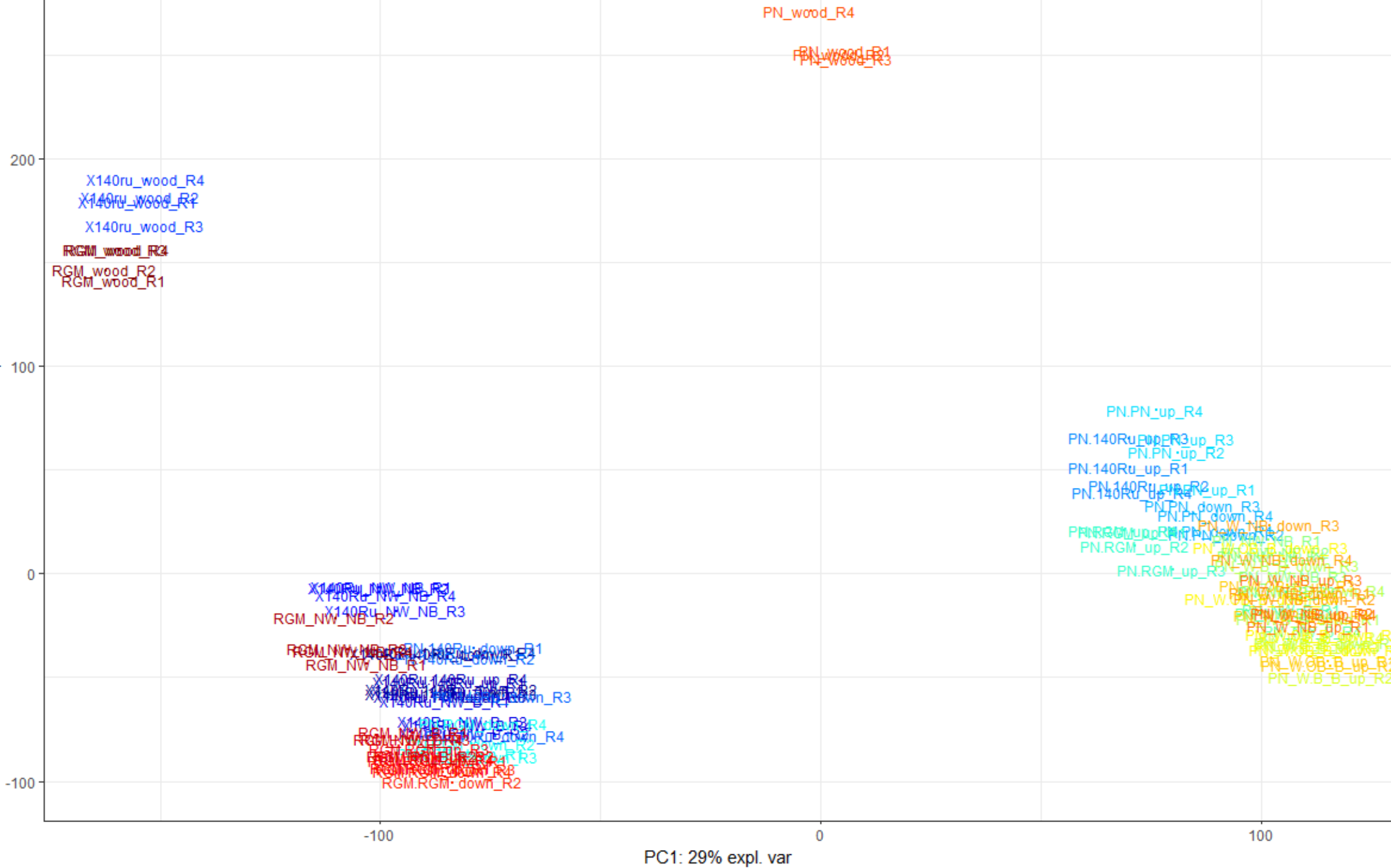

PC1: 29% expl. var

Supplement: Supplementary file 12 — Figure S12. Sample correlation and clustering heatmap. [file TPJ-122-0-s012.pdf]

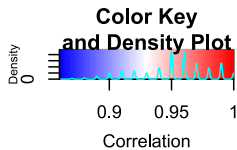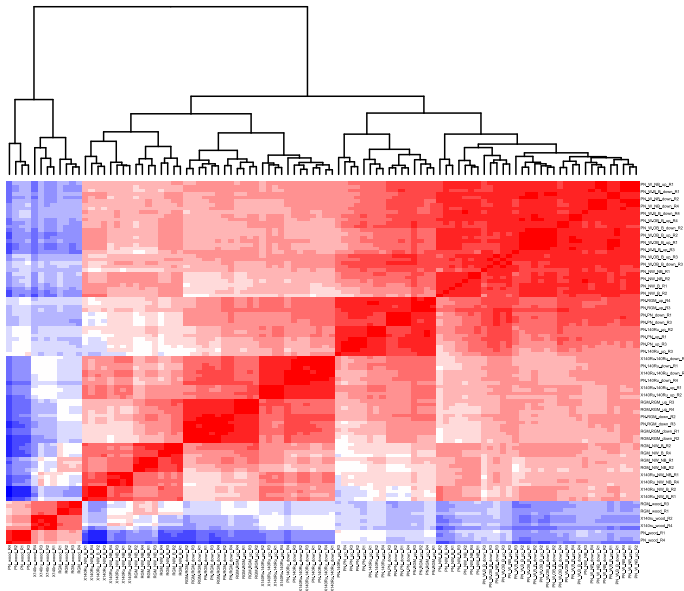

Supplement: Supplementary file 13 — Figure S13. Principle component (PC) analysis of samples. [file TPJ-122-0-s009.pdf]
