# Supplementary material for: Graft union formation involves interactions among bud signals, carbon availability, dormancy release, wound responses and non‐self‐communication in grapevine
Source: Plant J. 2025 Jun 11;122(5):e70244. doi: 10.1111/tpj.70244 (PMC12155988; doi:10.1111/tpj.70244)
Supplement: Supplementary file 3 — Figure S3. Heatmap of module‐metabolite. [file TPJ-122-0-s001.pdf]

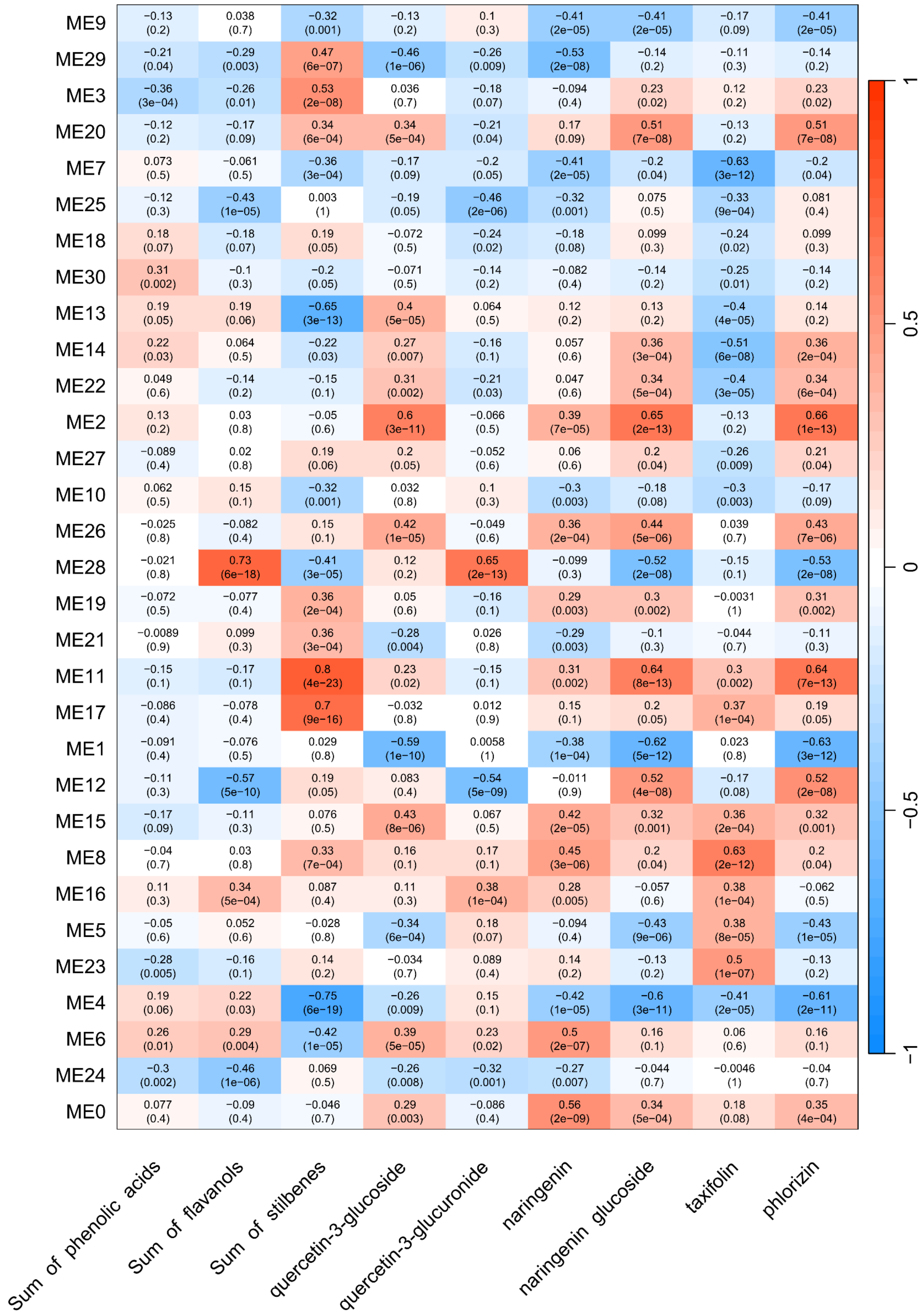
Sum of phenolic acids
Sum of flavanols
Sum of stilbenes
quercetin-3-glucoside
quercetin-3-glucuronide
naringenin
naringenin glucoside
taxifolin
phlorizin
ME9 −0.13 (0.2) 0.038 (0.7) −0.32 (0.001) −0.13 (0.2) 0.1 (0.3) −0.41 (2e−05) −0.41 (2e−05) −0.17 (0.09) −0.41 (2e−05)
ME29 −0.21 (0.04) −0.29 (0.003) 0.47 (6e−07) −0.46 (1e−06) −0.26 (0.009) −0.53 (2e−08) −0.14 (0.2) −0.11 (0.3) −0.14 (0.2)
ME3 −0.36 (3e−04) −0.26 (0.01) 0.53 (2e−08) 0.036 (0.7) −0.18 (0.07) −0.094 (0.4) 0.23 (0.02) 0.12 (0.2) 0.23 (0.02)
ME20 −0.12 (0.2) −0.17 (0.09) 0.34 (6e−04) 0.34 (5e−04) −0.21 (0.04) 0.17 (0.09) 0.51 (7e−08) −0.13 (0.2) 0.51 (7e−08)
ME7 0.073 (0.5) −0.061 (0.5) −0.36 (3e−04) −0.17 (0.09) −0.2 (0.05) −0.41 (2e−05) −0.2 (0.04) −0.63 (3e−12) −0.2 (0.04)
ME25 −0.12 (0.3) −0.43 (1e−05) 0.003 (1) −0.19 (0.05) −0.46 (2e−06) −0.32 (0.001) 0.075 (0.5) −0.33 (9e−04) 0.081 (0.4)
ME18 0.18 (0.07) −0.18 (0.07) 0.19 (0.05) −0.072 (0.5) −0.24 (0.02) −0.18 (0.08) 0.099 (0.3) −0.24 (0.02) 0.099 (0.3)
ME30 0.31 (0.002) −0.1 (0.3) −0.2 (0.05) −0.071 (0.5) −0.14 (0.2) −0.082 (0.4) −0.14 (0.2) −0.25 (0.01) −0.14 (0.2)
ME13 0.19 (0.05) 0.19 (0.06) −0.65 (3e−13) 0.4 (5e−05) 0.064 (0.5) 0.12 (0.2) 0.13 (0.2) −0.4 (4e−05) 0.14 (0.2)
ME14 0.22 (0.03) 0.064 (0.5) −0.22 (0.03) 0.27 (0.007) −0.16 (0.1) 0.057 (0.6) 0.36 (3e−04) −0.51 (6e−08) 0.36 (2e−04)
ME22 0.049 (0.6) −0.14 (0.2) −0.15 (0.1) 0.31 (0.002) −0.21 (0.03) 0.047 (0.6) 0.34 (5e−04) −0.4 (3e−05) 0.34 (6e−04)
ME2 0.13 (0.2) 0.03 (0.8) −0.05 (0.6) 0.6 (3e−11) −0.066 (0.5) 0.39 (7e−05) 0.65 (2e−13) −0.13 (0.2) 0.66 (1e−13)
ME27 −0.089 (0.4) 0.02 (0.8) 0.19 (0.06) 0.2 (0.05) −0.052 (0.6) 0.06 (0.6) 0.2 (0.04) −0.26 (0.009) 0.21 (0.04)
ME10 0.062 (0.5) 0.15 (0.1) −0.32 (0.001) 0.032 (0.8) 0.1 (0.3) −0.3 (0.003) −0.18 (0.08) −0.3 (0.003) −0.17 (0.09)
ME26 −0.025 (0.8) −0.082 (0.4) 0.15 (0.1) 0.42 (1e−05) −0.049 (0.6) 0.36 (2e−04) 0.44 (5e−06) 0.039 (0.7) 0.43 (7e−06)
ME28 −0.021 (0.8) 0.73 (6e−18) −0.41 (3e−05) 0.12 (0.2) 0.65 (2e−13) −0.099 (0.3) −0.52 (2e−08) −0.15 (0.1) −0.53 (2e−08)
ME19 −0.072 (0.5) −0.077 (0.4) 0.36 (2e−04) 0.05 (0.6) −0.16 (0.1) 0.29 (0.003) 0.3 (0.002) −0.0031 (1) 0.31 (0.002)
ME21 −0.0089 (0.9) 0.099 (0.3) 0.36 (3e−04) −0.28 (0.004) 0.026 (0.8) −0.29 (0.003) −0.1 (0.3) −0.044 (0.7) −0.11 (0.3)
ME11 −0.15 (0.1) −0.17 (0.1) 0.8 (4e−23) 0.23 (0.02) −0.15 (0.1) 0.31 (0.002) 0.64 (8e−13) 0.3 (0.002) 0.64 (7e−13)
ME17 −0.086 (0.4) −0.078 (0.4) 0.7 (9e−16) −0.032 (0.8) 0.012 (0.9) 0.15 (0.1) 0.2 (0.05) 0.37 (1e−04) 0.19 (0.05)
ME1 −0.091 (0.4) −0.076 (0.5) 0.029 (0.8) −0.59 (1e−10) 0.0058 (1) −0.38 (1e−04) −0.62 (5e−12) 0.023 (0.8) −0.63 (3e−12)
ME12 −0.11 (0.3) −0.57 (5e−10) 0.19 (0.05) 0.083 (0.4) −0.54 (5e−09) −0.011 (0.9) 0.52 (4e−08) −0.17 (0.08) 0.52 (2e−08)
ME15 −0.17 (0.09) −0.11 (0.3) 0.076 (0.5) 0.43 (8e−06) 0.067 (0.5) 0.42 (2e−05) 0.32 (0.001) 0.36 (2e−04) 0.32 (0.001)
ME8 −0.04 (0.7) 0.03 (0.8) 0.33 (7e−04) 0.16 (0.1) 0.17 (0.1) 0.45 (3e−06) 0.2 (0.04) 0.63 (2e−12) 0.2 (0.04)
ME16 0.11 (0.3) 0.34 (5e−04) 0.087 (0.4) 0.11 (0.3) 0.38 (1e−04) 0.28 (0.005) −0.057 (0.6) 0.38 (1e−04) −0.062 (0.5)
ME5 −0.05 (0.6) 0.052 (0.6) −0.028 (0.8) −0.34 (6e−04) 0.18 (0.07) −0.094 (0.4) −0.43 (9e−06) 0.38 (8e−05) −0.43 (1e−05)
ME23 −0.28 (0.005) −0.16 (0.1) 0.14 (0.2) −0.034 (0.7) 0.089 (0.4) 0.14 (0.2) −0.13 (0.2) 0.5 (1e−07) −0.13 (0.2)
ME4 0.19 (0.06) 0.22 (0.03) −0.75 (6e−19) −0.26 (0.009) 0.15 (0.1) −0.42 (1e−05) −0.6 (3e−11) −0.41 (2e−05) −0.61 (2e−11)
ME6 0.26 (0.01) 0.29 (0.004) −0.42 (1e−05) 0.39 (5e−05) 0.23 (0.02) 0.5 (2e−07) 0.16 (0.1) 0.06 (0.6) 0.16 (0.1)
ME24 −0.3 (0.002) −0.46 (1e−06) 0.069 (0.5) −0.26 (0.008) −0.32 (0.001) −0.27 (0.007) −0.044 (0.7) −0.0046 (1) −0.04 (0.7)
ME0 0.077 (0.4) −0.09 (0.4) −0.046 (0.7) 0.29 (0.003) −0.086 (0.4) 0.56 (2e−09) 0.34 (5e−04) 0.18 (0.08) 0.35 (4e−04)
1
0.5
0
−0.5
−1
