# Supplementary material for: Graft union formation involves interactions among bud signals, carbon availability, dormancy release, wound responses and non‐self‐communication in grapevine
Source: Plant J. 2025 Jun 11;122(5):e70244. doi: 10.1111/tpj.70244 (PMC12155988; doi:10.1111/tpj.70244)
Supplement: Supplementary file 6 — Figure S6. Genes differentially expressed and metabolites differentially accumulated in the wood during spring‐induced dormancy release. [file TPJ-122-0-s007.pdf]

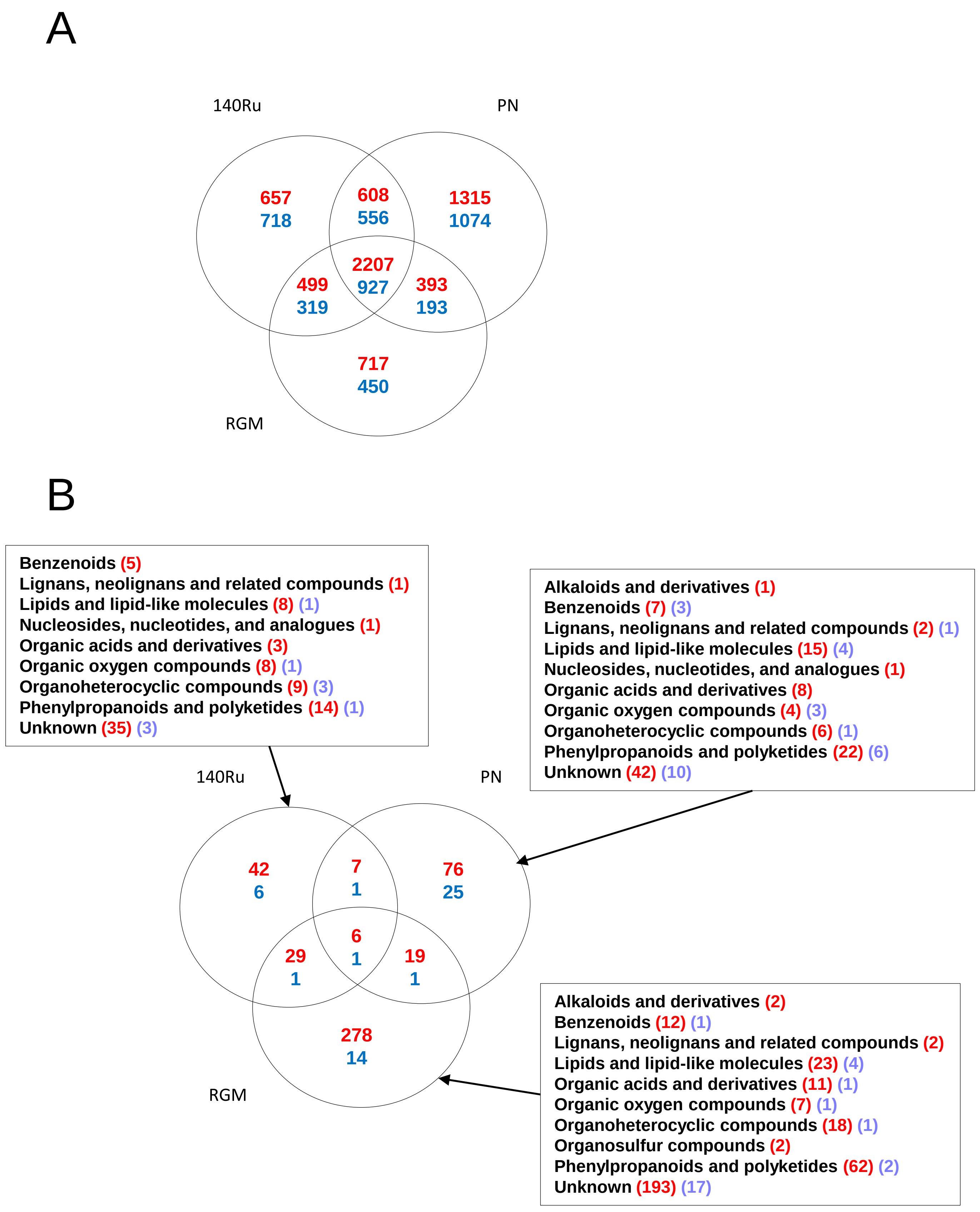

A
140Ru
PN
RGM
657
718
608
556
1315
1074
2207
927
499
319
393
193
717
450
B
Benzenoids (5)
Lignans, neolignans and related compounds (1)
Lipids and lipid-like molecules (8) (1)
Nucleosides, nucleotides, and analogues (1)
Organic acids and derivatives (3)
Organic oxygen compounds (8) (1)
Organoheterocyclic compounds (9) (3)
Phenylpropanoids and polyketides (14) (1)
Unknown (35) (3)
Alkaloids and derivatives (1)
Benzenoids (7) (3)
Lignans, neolignans and related compounds (2) (1)
Lipids and lipid-like molecules (15) (4)
Nucleosides, nucleotides, and analogues (1)
Organic acids and derivatives (8)
Organic oxygen compounds (4) (3)
Organoheterocyclic compounds (6) (1)
Phenylpropanoids and polyketides (22) (6)
Unknown (42) (10)
140Ru
PN
RGM
42
6
7
1
76
25
6
1
29
1
19
1
278
14
Alkaloids and derivatives (2)
Benzenoids (12) (1)
Lignans, neolignans and related compounds (2)
Lipids and lipid-like molecules (23) (4)
Organic acids and derivatives (11) (1)
Organic oxygen compounds (7) (1)
Organoheterocyclic compounds (18) (1)
Organosulfur compounds (2)
Phenylpropanoids and polyketides (62) (2)
Unknown (193) (17)
