# Supplementary material for: Graft union formation involves interactions among bud signals, carbon availability, dormancy release, wound responses and non‐self‐communication in grapevine
Source: Plant J. 2025 Jun 11;122(5):e70244. doi: 10.1111/tpj.70244 (PMC12155988; doi:10.1111/tpj.70244)
Supplement: Supplementary file 9 — Figure S9. Genes differentially expressed and metabolites differentially accumulated above and below the graft interface of grapevine homo‐grafts. [file TPJ-122-0-s018.pdf]

A

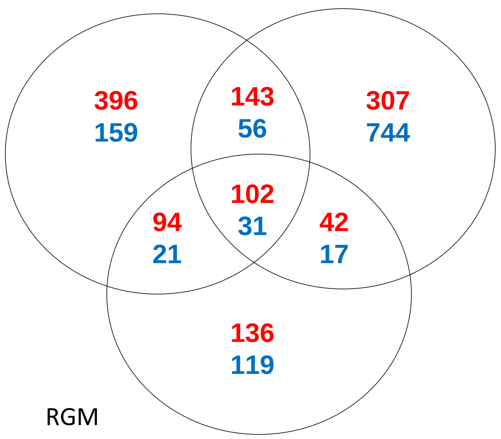

140Ru
PN
RGM
396
159
143
56
307
744
102
31
94
21
42
17
136
119

B

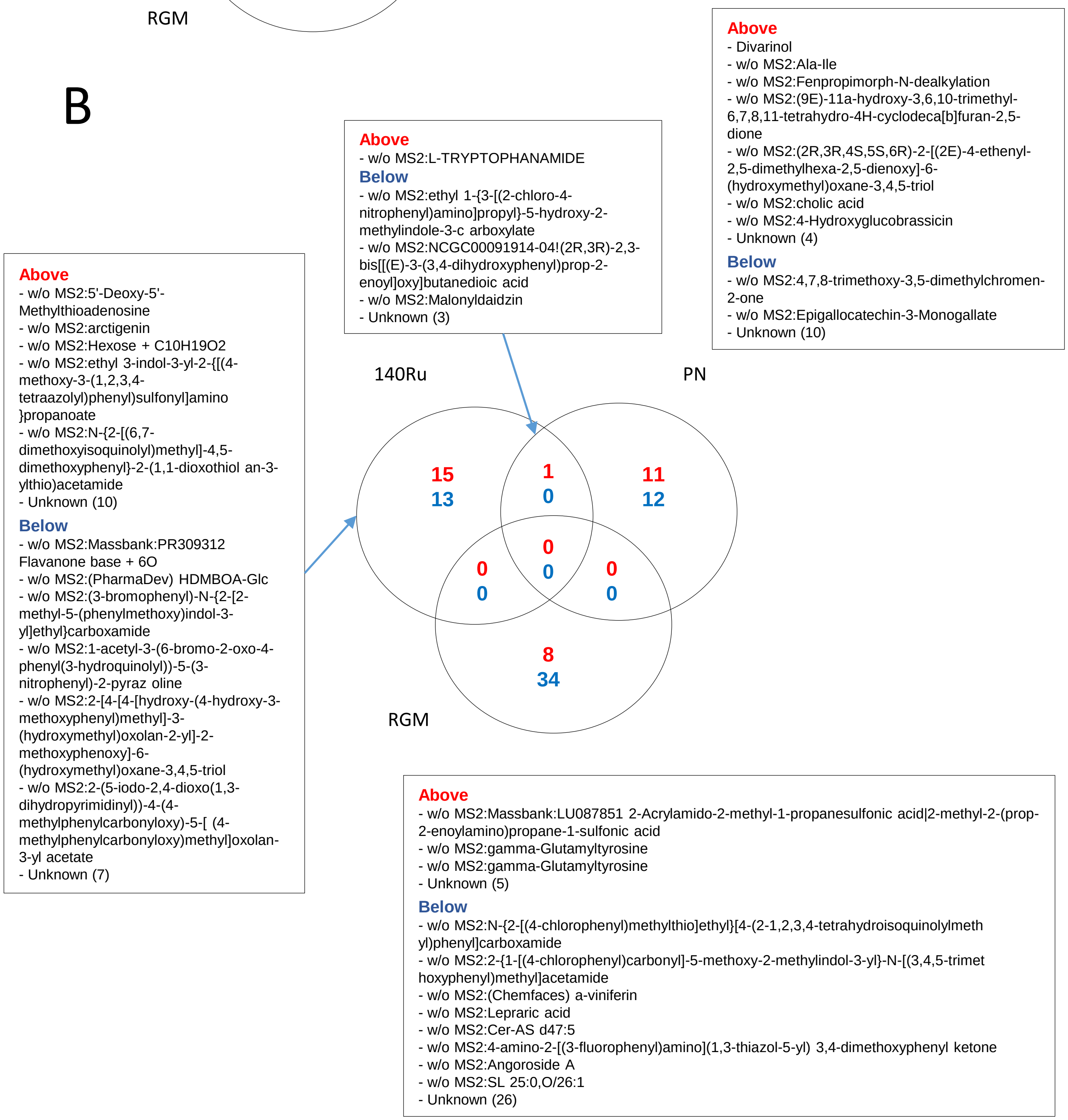

Above
- Divarinol
- w/o MS2:Ala-Ile
- w/o MS2:Fenpropimorph-N-dealkylation
- w/o MS2:(9E)-11a-hydroxy-3,6,10-trimethyl-6,7,8,11-tetrahydro-4H-cyclodeca[b]furan-2,5-dione
- w/o MS2:(2R,3R,4S,5S,6R)-2-[(2E)-4-ethenyl-2,5-dimethylhexa-2,5-dienoxy]-6-(hydroxymethyl)oxane-3,4,5-triol
- w/o MS2:cholic acid
- w/o MS2:4-Hydroxyglucobrassicin
- Unknown (4)
Below
- w/o MS2:4,7,8-trimethoxy-3,5-dimethylchromen-2-one
- w/o MS2:Epigallocatechin-3-Monogallate
- Unknown (10)
Above
- w/o MS2:L-TRYPTOPHANAMIDE
Below
- w/o MS2:ethyl 1-{3-[(2-chloro-4-nitrophenyl)amino]propyl}-5-hydroxy-2-methylindole-3-c arboxylate
- w/o MS2:NCGC00091914-04!(2R,3R)-2,3-bis[[(E)-3-(3,4-dihydroxyphenyl)prop-2-enoyl]oxy]butanedioic acid
- w/o MS2:Malonyldaidzin
- Unknown (3)
Above
- w/o MS2:5'-Deoxy-5'-Methylthioadenosine
- w/o MS2:arctigenin
- w/o MS2:Hexose + C10H19O2
- w/o MS2:ethyl 3-indol-3-yl-2-{[(4-methoxy-3-(1,2,3,4-tetraazolyl)phenyl)sulfonyl]amino}propanoate
- w/o MS2:N-{2-[(6,7-dimethoxyisoquinolyl)methyl]-4,5-dimethoxyphenyl}-2-(1,1-dioxothiol an-3-ylthio)acetamide
- Unknown (10)
Below
- w/o MS2:Massbank:PR309312 Flavanone base + 6O
- w/o MS2:(PharmaDev) HDMBOA-Glc
- w/o MS2:(3-bromophenyl)-N-{2-[2-methyl-5-(phenylmethoxy)indol-3-yl]ethyl}carboxamide
- w/o MS2:1-acetyl-3-(6-bromo-2-oxo-4-phenyl(3-hydroquinolyl))-5-(3-nitrophenyl)-2-pyraz oline
- w/o MS2:2-[4-[4-[hydroxy-(4-hydroxy-3-methoxyphenyl)methyl]-3-(hydroxymethyl)oxolan-2-yl]-2-methoxyphenoxy]-6-(hydroxymethyl)oxane-3,4,5-triol
- w/o MS2:2-(5-iodo-2,4-dioxo(1,3-dihydropyrimidinyl))-4-(4-methylphenylcarbonyloxy)-5-[(4-methylphenylcarbonyloxy)methyl]oxolan-3-yl acetate
- Unknown (7)
140Ru
PN
RGM
15
13
1
0
11
12
0
0
0
0
0
0
8
34
Above
- w/o MS2:Massbank:LU087851 2-Acrylamido-2-methyl-1-propanesulfonic acid|2-methyl-2-(prop-2-enoylamino)propane-1-sulfonic acid
- w/o MS2:gamma-Glutamyltyrosine
- w/o MS2:gamma-Glutamyltyrosine
- Unknown (5)
Below
- w/o MS2:N-{2-[(4-chlorophenyl)methylthio]ethyl}[4-(2-1,2,3,4-tetrahydroisoquinolylmethyl)phenyl]carboxamide
- w/o MS2:2-{1-[(4-chlorophenyl)carbonyl]-5-methoxy-2-methylindol-3-yl}-N-[(3,4,5-trimethoxyphenyl)methyl]acetamide
- w/o MS2:(Chemfaces) a-viniferin
- w/o MS2:Lepraric acid
- w/o MS2:Cer-AS d47:5
- w/o MS2:4-amino-2-[(3-fluorophenyl)amino](1,3-thiazol-5-yl) 3,4-dimethoxyphenyl ketone
- w/o MS2:Angoroside A
- w/o MS2:SL 25:0,O/26:1
- Unknown (26)
