# Supplementary material for: Graft union formation involves interactions among bud signals, carbon availability, dormancy release, wound responses and non‐self‐communication in grapevine
Source: Plant J. 2025 Jun 11;122(5):e70244. doi: 10.1111/tpj.70244 (PMC12155988; doi:10.1111/tpj.70244)
Supplement: Supplementary file 27 — Table S1. MapMan BINs enriched in the genes presented in ME17. Table S2. Gene Ontology (GO) terms enriched in the genes present in ME17. Table S3. Gene Ontology (GO) terms enriched in the genes present in ME11. Table S4. MapMan BINs enriched in the genes presented in ME11. Table S5. MapMan BINs enriched in the 848 genes more highly expressed in the rootstock than scion 14 days after grafting. Table S6. MapMan BINs enriched in the 594 genes more highly expressed in the scion than rootstock 14 days after grafting. Table S7. MapMan BINs enriched in the 364 genes more highly expressed in response to wounding than grafting in both scions and rootstocks. Table S8. MapMan BINs enriched in the 477 genes more highly expressed in response to grafting than wounding in both scions and rootstocks. Table S9. MapMan BINs enriched in the 59 genes more highly expressed in scions of homo‐ than hetero‐grafts. Table S10. Gene Ontology (GO) terms enriched in the 175 genes more highly expressed in scions of homo‐ than hetero‐grafts. Table S11. MapMan BINs enriched in the 175 genes more highly expressed in scions of homo‐ than hetero‐grafts. Table S12. Grafts and cuttings used in this study. Table S13. Parameters for the analysis of metabolites by HPLC‐QqQ in MRM mode. [file TPJ-122-0-s023.docx]

Table S1. MapMan BINs enriched in the genes presented in ME17, a cluster of genes specifically up regulated in response to grafting.

| **BIN** | **BIN Name** | **Adjusted p value** |
| --- | --- | --- |
| 21.9 | Cell wall organisation.cutin and suberin | 5.41E-05 |
| 21.9.1 | Cell wall organisation.cutin and suberin.cuticular lipid formation | 6.62E-03 |
| 21.9.1.5 | Cell wall organisation.cutin and suberin.cuticular lipid formation.monoacylglyceryl ester monomer biosynthesis | 4.26E-03 |
| 5 | Lipid metabolism | 8.89E-03 |
| 5.2 | Lipid metabolism.glycerolipid metabolism | 4.64E-02 |
| 5.2.1 | Lipid metabolism.glycerolipid metabolism.phosphatidate biosynthesis | 6.62E-03 |
| 5.2.1.2 | Lipid metabolism.glycerolipid metabolism.phosphatidate biosynthesis.endoplasmic reticulum phosphatidate biosynthesis | 8.86E-03 |
| 5.2.1.2.2 | Lipid metabolism.glycerolipid metabolism.phosphatidate biosynthesis.endoplasmic reticulum phosphatidate biosynthesis.glycerol-3-phosphate acyltransferase (GPAT4-8) | 1.06E-02 |
| 18.4.25.2.1 | Protein modification.phosphorylation.protein serine/threonine phosphatase superfamily.PPM/PP2C Mn/Mg-dependent phosphatase families.clade A phosphatase | 6.20E-03 |
| 24 | Solute transport | 5.01E-03 |
| 24.1.3 | Solute transport.primary active transport.ABC superfamily | 4.39E-03 |
| 24.1.3.2 | Solute transport.primary active transport.ABC superfamily.ABC2 family | 2.76E-02 |
| 24.1.3.2.2 | Solute transport.primary active transport.ABC superfamily.ABC2 family.subfamily ABCG transporter | 1.62E-02 |

Table S2. Gene Ontology (GO) terms enriched in the genes present in ME17, a cluster of genes specifically up regulated in response to grafting.

| **GO identifier** | **GO description** | **Adjusted p-value** |
| --- | --- | --- |
| GO:0055085 | transmembrane transport | 5.30E-05 |
| GO:0009737 | response to abscisic acid | 0.00015 |
| GO:0006869 | lipid transport | 0.00089 |
| GO:0016311 | dephosphorylation | 0.00327 |
| GO:0006633 | fatty acid biosynthetic process | 0.01162 |
| GO:0042744 | hydrogen peroxide catabolic process | 0.04503 |
| GO:0035556 | intracellular signal transduction | 0.04858 |

Table S3. Gene Ontology (GO) terms enriched in the genes present in ME11, a cluster of genes up regulated in response to wounding and grafting.

| **GO identifier** | **GO term description** | **Adjusted p value** |
| --- | --- | --- |
| GO:0010951 | negative regulation of endopeptidase activity | 2.90E-05 |
| GO:0042744 | hydrogen peroxide catabolic process | 0.00017 |
| GO:0002229 | defense response to oomycetes | 0.00066 |
| GO:0007178 | transmembrane receptor protein serine/threonine kinase signaling pathway | 0.0008 |
| GO:0006979 | response to oxidative stress | 0.0018 |
| GO:0098869 | cellular oxidant detoxification | 0.00196 |
| GO:0010411 | xyloglucan metabolic process | 0.01149 |
| GO:0055085 | transmembrane transport | 0.0115 |
| GO:0042742 | defense response to bacterium | 0.0138 |
| GO:0048544 | recognition of pollen | 0.02957 |
| GO:0006468 | protein phosphorylation | 0.03368 |
| GO:0006807 | nitrogen compound metabolic process | 0.04241 |
| GO:0042546 | cell wall biogenesis | 0.04858 |

Table S4. MapMan BINs enriched in the genes presented in ME11, a cluster of genes up regulated in response to wounding and grafting.

| **BIN** | **BIN Name** | **Adjusted p value** |
| --- | --- | --- |
| 2 | Cellular respiration | 8.42E-04 |
| 2.4 | Cellular respiration.oxidative phosphorylation | 2.69E-02 |
| 50 | Enzyme classification | 1.71E-04 |
| 50.1 | Enzyme classification.EC_1 oxidoreductases | 1.15E-02 |
| 50.1.1 | Enzyme classification.EC_1 oxidoreductases.EC_1.1 oxidoreductase acting on CH-OH group of donor | 8.50E-03 |
| 11.2.3 | Phytohormone action.auxin.conjugation and degradation | 1.30E-02 |
| 11.8.4 | Phytohormone action.salicylic acid.transport | 1.14E-02 |
| 19.4.6 | Protein homeostasis.proteolysis.protease inhibitor activities | 1.44E-03 |
| 9.2 | Secondary metabolism.phenolics | 1.30E-03 |
| 9.2.2 | Secondary metabolism.phenolics.flavonoid biosynthesis | 3.97E-03 |

Table S5. MapMan BINs enriched in the 848 genes more highly expressed in the rootstock than scion in homografts 14 d after grafting.

Fold change calculated as the ratio of expression between the rootstock and scion of homografts of *Vitis vinifera* cv. Pinot Noir 14 d after grafting, genes considered differentially expressed with an absolute log₂-fold change > 1.5 and the adjusted p-value (False discovery rate) < 0.05.

| **BIN** | **BIN Name** | **Adjusted p value** |
| --- | --- | --- |
| 13 | Cell division | 8.39E-45 |
| 13.2 | Cell division.cell cycle organisation | 7.51E-23 |
| 13.2.1 | Cell division.cell cycle organisation.cell cycle control | 2.12E-08 |
| 13.2.1.1 | Cell division.cell cycle organisation.cell cycle control.CYCLIN regulatory protein activities | 2.99E-07 |
| 13.2.1.1.2 | Cell division.cell cycle organisation.cell cycle control.CYCLIN regulatory protein activities.regulatory protein (CYCB) | 1.04E-08 |
| 13.2.2 | Cell division.cell cycle organisation.chromatin condensation | 2.62E-08 |
| 13.2.2.1 | Cell division.cell cycle organisation.chromatin condensation.condensin I/II complex | 2.62E-08 |
| 13.2.2.1.4 | Cell division.cell cycle organisation.chromatin condensation.condensin I/II complex.condensin-II-specific components | 1.09E-02 |
| 13.2.2.1.3 | Cell division.cell cycle organisation.chromatin condensation.condensin I/II complex.condensin-I-specific components | 4.90E-02 |
| 13.2.5 | Cell division.cell cycle organisation.sister chromatid separation | 6.10E-04 |
| 13.2.5.6 | Cell division.cell cycle organisation.sister chromatid separation.meiosis II spindle orientation | 3.15E-02 |
| 13.4 | Cell division.cytokinesis | 9.09E-08 |
| 13.4.3 | Cell division.cytokinesis.phragmoplast microtubule organization | 3.91E-09 |
| 13.4.3.7 | Cell division.cytokinesis.phragmoplast microtubule organization.regulatory protein (Kinesin-12) of phragmoplast integrity | 3.15E-02 |
| 13.1 | Cell division.DNA replication | 2.22E-12 |
| 13.1.3 | Cell division.DNA replication.elongation | 3.43E-03 |
| 13.1.3.1 | Cell division.DNA replication.elongation.DNA polymerase alpha complex | 3.71E-03 |
| 13.1.2 | Cell division.DNA replication.initiation | 7.43E-03 |
| 13.1.1 | Cell division.DNA replication.preinitiation | 7.65E-12 |
| 13.1.1.2 | Cell division.DNA replication.preinitiation.MCM replicative DNA helicase complex | 1.08E-08 |
| 13.3 | Cell division.meiotic recombination | 4.07E-03 |
| 13.3.6 | Cell division.meiotic recombination.meiotic exit | 7.56E-04 |
| 13.3.6.1 | Cell division.meiotic recombination.meiotic exit.regulatory protein (MS5/TDM1) of meiotic exit | 3.15E-02 |
| 21 | Cell wall organisation | 2.64E-04 |
| 21.3 | Cell wall organisation.pectin | 1.60E-03 |
| 12 | Chromatin organisation | 1.03E-05 |
| 12.1 | Chromatin organisation.chromatin structure | 1.07E-09 |
| 12.1.1 | Chromatin organisation.chromatin structure.DNA wrapping | 2.57E-08 |
| 12.1.1.3 | Chromatin organisation.chromatin structure.DNA wrapping.histone (H3) | 3.66E-04 |
| 20 | Cytoskeleton organisation | 1.72E-13 |
| 20.1 | Cytoskeleton organisation.microtubular network | 2.12E-20 |
| 20.1.3 | Cytoskeleton organisation.microtubular network.Kinesin microtubule-based motor protein activities | 1.78E-16 |
| 20.1.3.10 | Cytoskeleton organisation.microtubular network.Kinesin microtubule-based motor protein activities.motor protein (Kinesin-12) | 1.82E-05 |
| 20.1.3.4 | Cytoskeleton organisation.microtubular network.Kinesin microtubule-based motor protein activities.motor protein (Kinesin-5) | 3.15E-02 |
| 20.1.5 | Cytoskeleton organisation.microtubular network.microtubule dynamics | 1.31E-02 |
| 50 | Enzyme classification | 6.56E-07 |
| 50.3 | Enzyme classification.EC_3 hydrolases | 2.39E-04 |
| 50.3.2 | Enzyme classification.EC_3 hydrolases.EC_3.2 glycosylase | 2.97E-03 |
| 11.4.3 | Phytohormone action.cytokinin.conjugation and degradation | 2.83E-03 |
| 11.4.3.2 | Phytohormone action.cytokinin.conjugation and degradation.cytokinin dehydrogenase (CKX) | 1.25E-02 |

Table S6. MapMan BINs enriched in the 594 genes more highly expressed in the scion than rootstock 14 d after grafting.

Fold change calculated as the ratio of expression between the scion and rootstock of homografts of *Vitis vinifera* cv. Pinot Noir 14 d after grafting, genes considered differentially expressed with an absolute log₂-fold change > 1.5 and the adjusted p-value (False discovery rate) < 0.05.

| **BIN** | **BIN Name** | **Adjusted p value** |
| --- | --- | --- |
| 3.4 | Carbohydrate metabolism.oligosaccharide metabolism | 1.21E-06 |
| 3.4.1 | Carbohydrate metabolism.oligosaccharide metabolism.galactinol synthase | 2.20E-06 |
| 21 | Cell wall organisation | 5.62E-07 |
| 21.6 | Cell wall organisation.lignin | 8.32E-05 |
| 21.6.2 | Cell wall organisation.lignin.monolignol conjugation and polymerization | 1.98E-07 |
| 21.6.2.2 | Cell wall organisation.lignin.monolignol conjugation and polymerization.lignin laccase | 1.79E-08 |
| 11 | Phytohormone action | 3.26E-10 |
| 11.2 | Phytohormone action.auxin | 4.75E-02 |
| 11.2.2 | Phytohormone action.auxin.perception and signal transduction | 2.25E-02 |
| 11.2.2.2 | Phytohormone action.auxin.perception and signal transduction.transcriptional repressor (IAA/AUX) | 8.76E-04 |
| 11.4 | Phytohormone action.cytokinin | 2.81E-02 |
| 11.1 | Phytohormone action.signalling peptides | 1.45E-04 |
| 11.10.2.2 | Phytohormone action.signalling peptides.CRP (cysteine-rich-peptide) category.STIG1-peptide activity | 4.76E-04 |
| 11.10.2.2.1 | Phytohormone action.signalling peptides.CRP (cysteine-rich-peptide) category.STIG1-peptide activity.STIG1/GRI-precursor polypeptide | 2.81E-04 |
| 11.10.1 | Phytohormone action.signalling peptides.NCRP (non-cysteine-rich-peptide) category | 8.37E-03 |
| 18.4.1.15 | Protein modification.phosphorylation.TKL protein kinase superfamily.protein kinase (LRR-XV) | 1.19E-02 |
| 18.4.1.25 | Protein modification.phosphorylation.TKL protein kinase superfamily.protein kinase (WAK/WAKL) | 4.54E-02 |
| 24 | Solute transport | 4.01E-04 |
| 24.2 | Solute transport.carrier-mediated transport | 3.90E-05 |
| 24.2.1.5 | Solute transport.carrier-mediated transport.DMT superfamily.solute transporter (UmamiT) | 8.60E-03 |

Table S7. MapMan BINs enriched in the 364 genes more highly expressed in response to wounding than grafting in both scions and rootstocks.

Fold change calculated as the ratio of expression between the wounding and grafting of *Vitis vinifera* cv. Pinot noir 14 d after treatment, genes considered differentially expressed with an absolute log₂-fold change > 1.5 and the adjusted p-value (False discovery rate) < 0.05.

| **BIN** | **BIN Name** | **Adjusted p value** |
| --- | --- | --- |
| 21.6.1.4 | Cell wall organisation.lignin.monolignol biosynthesis.caffeoyl-CoA 3-O-methyltransferase (CCoA-OMT) | 3.93E-02 |
| 26.3 | External stimuli response.gravity | 3.27E-03 |
| 26.3.1 | External stimuli response.gravity.sensing and signalling | 3.27E-03 |
| 19.4.2.1.1 | Protein homeostasis.proteolysis.serine-type peptidase activities.S8-class protease (subtilisin) families.protease (SBT1) | 3.20E-02 |
| 15.5 | RNA biosynthesis.transcriptional regulation | 5.93E-03 |
| 24 | Solute transport | 9.67E-05 |
| 24.2 | Solute transport.carrier-mediated transport | 2.47E-04 |
| 24.2.2.9 | Solute transport.carrier-mediated transport.MFS superfamily.anion transporter (NRT1/PTR) | 9.29E-03 |

Table S8. MapMan BINs enriched in the 477 genes more highly expressed in response to grafting than wounding in both scions and rootstocks.

Fold change calculated as the ratio of expression between the grafting and wounding of *Vitis vinifera* cv. Pinot noir 14 d after treatment, genes considered differentially expressed with an absolute log₂-fold change > 1.5 and the adjusted p-value (False discovery rate) < 0.05.

| **BIN** | **BIN Name** | **Adjusted p value** |
| --- | --- | --- |
| 3.4 | Carbohydrate metabolism.oligosaccharide metabolism | 4.83E-03 |
| 21.9 | Cell wall organisation.cutin and suberin | 9.05E-03 |
| 50 | Enzyme classification | 1.40E-09 |
| 50.1 | Enzyme classification.EC_1 oxidoreductases | 1.88E-02 |
| 50.2 | Enzyme classification.EC_2 transferases | 9.79E-04 |
| 50.2.4 | Enzyme classification.EC_2 transferases.EC_2.4 glycosyltransferase | 4.86E-04 |
| 11 | Phytohormone action | 6.38E-03 |
| 11.7 | Phytohormone action.jasmonic acid | 3.31E-05 |
| 11.7.1 | Phytohormone action.jasmonic acid.biosynthesis | 9.12E-03 |
| 19.4.6 | Protein homeostasis.proteolysis.protease inhibitor activities | 1.89E-02 |
| 19.4.6.2 | Protein homeostasis.proteolysis.protease inhibitor activities.Kunitz protease inhibitor | 2.09E-02 |
| 19.2.2.1.4.2 | Protein homeostasis.ubiquitin-proteasome system.ubiquitin-fold protein conjugation.ubiquitin conjugation (ubiquitylation).ubiquitin-ligase E3 activities.U-Box E3 ligase activities | 7.08E-03 |
| 18.4.25.2 | Protein modification.phosphorylation.protein serine/threonine phosphatase superfamily.PPM/PP2C Mn/Mg-dependent phosphatase families | 9.05E-03 |
| 18.4.25.2.1 | Protein modification.phosphorylation.protein serine/threonine phosphatase superfamily.PPM/PP2C Mn/Mg-dependent phosphatase families.clade A phosphatase | 5.93E-03 |
| 15.5.22 | RNA biosynthesis.transcriptional regulation.WRKY transcription factor activity | 1.98E-04 |
| 15.5.22.1 | RNA biosynthesis.transcriptional regulation.WRKY transcription factor activity.transcription factor (WRKY) | 7.98E-05 |
| 24 | Solute transport | 2.23E-06 |
| 24.1 | Solute transport.primary active transport | 3.22E-03 |
| 24.1.3 | Solute transport.primary active transport.ABC superfamily | 3.46E-02 |
| 24.1.2.2 | Solute transport.primary active transport.P-type ATPase superfamily.P2 family | 2.61E-02 |
| 24.1.2.2.2 | Solute transport.primary active transport.P-type ATPase superfamily.P2 family.P2B-type calcium cation-transporting ATPase (ACA) | 1.11E-02 |
| 22.4.1.1 | Vesicle trafficking.exocytic trafficking.Exocyst complex.component EXO70 | 1.58E-02 |

Table S9. MapMan BINs enriched in the 59 genes more highly expressed in response to hetero- than homo-grafting.

*Vitis vinifera* cv. Pinot noir scions were either homografted or hetero-grafted with *V. rupestris x V. berlandieri* cv. 140 Ruggeri or *V. riparia* cv. Gloire de Montpellier. Fold change calculated as the ratio of expression between the hetero- and homo-grafts 14 d after grafting, genes considered differentially expressed with an absolute log₂-fold change > 1.5 and the adjusted p-value (False discovery rate) < 0.05. 59 genes were common to both comparisons.

| **BIN** | **BIN Name** | **Adjusted p value** |
| --- | --- | --- |
| 50.3 | Enzyme classification.EC_3 hydrolases | 1.06E-02 |
| 1.1.1.1 | Photosynthesis.photophosphorylation.photosystem II.LHC-II complex | 2.57E-03 |
| 15.5 | RNA biosynthesis.transcriptional regulation | 1.64E-02 |
| 15.5.30 | RNA biosynthesis.transcriptional regulation.transcription factor (bHLH) | 6.59E-04 |

Table S10. Gene Ontology (GO) terms enriched in the 175 genes more highly expressed in scions of homo- than hetero-grafts. *Vitis vinifera* cv. Pinot noir scions were either homografted or hetero-grafted with 140Ru, *V. rupestris x V. berlandieri* cv. 140 Ruggeri or *V. riparia* cv. Gloire de Montpellier. Fold change calculated as the ratio of expression between the homo- and hetero-grafts 14 d after grafting, genes considered differentially expressed with an absolute log₂-fold change > 1.5 and the adjusted p-value (False discovery rate) < 0.05. 175 genes were common to both comparisons.

| **GO identifier** | **GO Term** | **Adjusted p value** |
| --- | --- | --- |
| GO:0010411 | xyloglucan metabolic process | 9.40E-12 |
| GO:0042546 | cell wall biogenesis | 3.00E-07 |
| GO:0006355 | regulation of DNA-templated transcription | 9.50E-07 |
| GO:0071555 | cell wall organization | 6.00E-06 |
| GO:0070588 | calcium ion transmembrane transport | 2.30E-05 |
| GO:0050790 | regulation of catalytic activity | 0.0021 |

Table S11. MapMan BINs enriched in the 175 genes more highly expressed in scions of homo- than hetero-grafts. *Vitis vinifera* cv. Pinot noir scions were either homografted or hetero-grafted with 140Ru, *V. rupestris x V. berlandieri* cv. 140 Ruggeri or *V. riparia* cv. Gloire de Montpellier. Fold change calculated as the ratio of expression between the homo- and hetero-grafts 14 d after grafting, genes considered differentially expressed with an absolute log₂-fold change > 1.5 and the adjusted p-value (False discovery rate) < 0.05. 175 genes were common to both comparisons.

| **BIN** | **BIN Name** | **Adjusted p value** |
| --- | --- | --- |
| 7.12.1.2 | Coenzyme metabolism.tetrapyrrole biosynthesis.5-aminolevulinic acid formation.glutamyl-tRNA reductase activity | 4.82E-01 |
| 50.2.4 | Enzyme classification.EC_2 transferases.EC_2.4 glycosyltransferase | 7.51E-03 |
| 26 | External stimuli response | 2.46E-03 |
| 26.9 | External stimuli response.pathogen | 9.43E-04 |
| 26.9.2 | External stimuli response.pathogen.effector-triggered immunity (ETI) network | 4.26E-03 |
| 26.9.2.1 | External stimuli response.pathogen.effector-triggered immunity (ETI) network.TNL-mediated effector-triggered immunity | 6.72E-03 |
| 26.4 | External stimuli response.temperature | 1.90E-02 |
| 26.4.3 | External stimuli response.temperature.cold response | 8.17E-04 |
| 26.4.3.4 | External stimuli response.temperature.cold response.ICE-CBF cold acclimation transcriptional cascade | 4.10E-05 |
| 26.4.3.4.2 | External stimuli response.temperature.cold response.ICE-CBF cold acclimation transcriptional cascade.transcription factor (CBF/DREB1) | 2.41E-04 |
| 5.2.10.2 | Lipid metabolism.glycerolipid metabolism.glycerolipid degradation.phospholipase activities | 4.07E-01 |
| 5.2.10.2.1 | Lipid metabolism.glycerolipid metabolism.glycerolipid degradation.phospholipase activities.phospholipase A1 activities | 4.33E-03 |
| 27 | Multi-process regulation | 1.41E-06 |
| 27.8 | Multi-process regulation.calcium-dependent signalling | 2.92E-14 |
| 27.8.2 | Multi-process regulation.calcium-dependent signalling.calcium sensor (CML) | 8.14E-15 |
| 19.2.2.1.4 | Protein homeostasis.ubiquitin-proteasome system.ubiquitin-fold protein conjugation.ubiquitin conjugation (ubiquitylation).ubiquitin-ligase E3 activities | 4.83E-01 |
| 19.2.2.1.4.2 | Protein homeostasis.ubiquitin-proteasome system.ubiquitin-fold protein conjugation.ubiquitin conjugation (ubiquitylation).ubiquitin-ligase E3 activities.U-Box E3 ligase activities | 2.65E-06 |
| 19.2.2.1.4.2.2 | Protein homeostasis.ubiquitin-proteasome system.ubiquitin-fold protein conjugation.ubiquitin conjugation (ubiquitylation).ubiquitin-ligase E3 activities.U-Box E3 ligase activities.group-III ligase | 5.68E-04 |
| 15 | RNA biosynthesis | 5.30E-04 |
| 15.5 | RNA biosynthesis.transcriptional regulation | 1.26E-05 |
| 15.5.7 | RNA biosynthesis.transcriptional regulation.AP2/ERF transcription factor superfamily | 7.72E-11 |
| 15.5.7.2 | RNA biosynthesis.transcriptional regulation.AP2/ERF transcription factor superfamily.transcription factor (DREB) | 1.10E-11 |
| 15.5.22 | RNA biosynthesis.transcriptional regulation.WRKY transcription factor activity | 3.08E-03 |
| 15.5.22.1 | RNA biosynthesis.transcriptional regulation.WRKY transcription factor activity.transcription factor (WRKY) | 3.48E-02 |
| 24.1.2 | Solute transport.primary active transport.P-type ATPase superfamily | 3.63E-03 |
| 24.1.2.2 | Solute transport.primary active transport.P-type ATPase superfamily.P2 family | 2.29E-05 |
| 24.1.2.2.2 | Solute transport.primary active transport.P-type ATPase superfamily.P2 family.P2B-type calcium cation-transporting ATPase (ACA) | 4.27E-04 |

Table S12: Grafts and cutting used in this study with their Vitis International Variety Catalogue numbers in brackets.

| Abbreviation | Scion genotype | Rootstock genotype |
| --- | --- | --- |
| *Heterografts* |  |  |
| PN/RGM | *Vitis vinifera* cv. Pinot Noir clone 115 (9279) | *V. riparia Michaux* cv. Riparia Gloire de Montpellier clone 1030 (4824) |
| PN/140Ru | *V. vinifera* cv. Pinot Noir clone 115 (9279) | *V. berlandieri* x *V. rupestris* cv. 140 Ruggeri clone 265 (10351) |
| *Homografts* |  |  |
| PN/PN | *V. vinifera* cv. Pinot Noir clone 115 (9279) | *V. vinifera* cv. Pinot Noir clone 115 (9279) |
| RGM/RGM | *Vitis riparia Michaux* cv. Riparia Gloire de Montpellier clone 1030 (4824) | *Vitis riparia Michaux* cv. Riparia Gloire de Montpellier clone 1030 (4824) |
| 140Ru/140Ru | *V. berlandieri* x *V. rupestris* cv. 140 Ruggeri clone 265 (10351) | *V. berlandieri* x *V. rupestris* cv. 140 Ruggeri clone 265 (10351) |
| *Cuttings* |  |  |
| PN | *V. vinifera* cv. Pinot Noir clone 115 (9279)  *V. berlandieri* x *V. rupestris* cv. 140 Ruggeri clone 265 (10351)  *Vitis riparia Michaux* cv. Riparia Gloire de Montpellier clone 1030 (4824) | |
| 140Ru |  |  |
| RGM |  |  |

Table S13: Mass of precursor ion (*Precursor Ion*), mass of one of the fragments used for quantification (*Quantifier*), retention time (*Rt*) and ion polarity of the 39 secondary metabolites analysed by HPLC-QqQ in MRM mode.

|  | *Precusor Ion* | *Quantifier* | *Rt (min)* | | *Ion Polarity* |  |
| --- | --- | --- | --- | --- | --- | --- |
| *Phenolic acids* |  |  |  |  | | |
| gallic acid | 169 | 125 | 4.2 | Negative | | |
| caftaric acid | 311 | 149 | 8.3 | Negative | | |
| coutaric acid | 295 | 163 | 12.6 | Negative | | |
| *Flavonols* |  |  |  |  | | |
| quercetin 3 glucoside | 463 | 301 | 15.5 | Negative | | |
| quercetin 3 glucuronide | 477 | 301 | 15.4 | Negative | | |
| *Flavanols* |  |  |  |  | | |
| catechin | 289 | 109 | 10 | Negative | | |
| Epicatechin | 289 | 109 | 11.8 | Negative | | |
| epicatechin gallate | 441 | 289.1 | 15.2 | Negative | | |
| epigallocatechin | 305 | 125 | 9.2 | Negative | | |
| epigallocatechin gallate | 457 | 169 | 12.1 | Negative | | |
| gallocatechin | 305 | 125 | 7.5 | Negative | | |
| B1 | 577 | 289 | 9.4 | Negative | | |
| B2 | 577 | 289 | 11.1 | Negative | | |
| B3 | 577 | 289 | 9.6 | Negative | | |
| B4 | 577 | 289 | 10.5 | Negative | | |
| C1 | 865 | 125 | 13 | Negative | | |
| *Stilbenes* |  |  |  |  | | |
| *trans*-resveratrol | 229 | 107 | 17.9 | Positive | | |
| *trans*-piceid | 389 | 227 | 14.7 | Negative | | |
| *cis*-piceid | 389 | 227 | 17.2 | Negative | | |
| *trans*-piceatannol | 243 | 159 | 16.2 | Negative | | |
| *trans*-astringin | 405 | 243 | 12.5 | Negative | | |
| *cis*-astringin | 405 | 243 | 15.5 | Negative | | |
| *trans*-ε-viniferin | 455 | 107 | 21.4 | Positive | | |
| *trans-*ω-viniferin | 455 | 107 | 22.4 | Positive | | |
| *trans-*δ*-*viniferin | 455 | 361 | 23 | Positive | | |
| pallidol | 455 | 361 | 17.6 | Positive | | |
| parthenocisin A | 455 | 361 | 18.2 | Positive | | |
| ampelopsin A | 471 | 453 | 16.2 | Positive | | |
| vitisinol C | 429 | 107 | 21.4 | Positive | | |
| α-viniferin | 679 | 359 | 22.6 | Positive | | |
| miyabenol C | 681 | 575 | 21.9 | Positive | | |
| hopeaphenol | 908 | 359 | 19.6 | Positive | | |
| isohopeaphenol | 908 | 359 | 20.0 | Positive | | |
| r2-viniferin | 908 | 559 | 22.4 | Positive | | |
| r-viniferin | 908 | 559 | 23.5 | Positive | | |
| *Others* |  |  |  |  | | |
| naringenin | 271 | 119.1 | 21.3 | Negative | | |
| naringenin glucoside | 435 | 273 | 19.7 | Positive | | |
| taxifolin | 303 | 125.1 | 15.4 | Negative | | |
| phlorizin | 435 | 273 | 16.7 | Positive | | |
